# Supplementary material for: Tolerance to soil acidity of soybean (Glycine max L.) genotypes under field conditions Southwestern Ethiopia
Source: PLoS One. 2022 Sep 15;17(9):e0272924. doi: 10.1371/journal.pone.0272924 (PMC9477287; doi:10.1371/journal.pone.0272924)
Supplement: S1 Table — (DOC) [file pone.0272924.s001.doc]

**Supporting information" files.**

**Raw data**

| | rep | | --- | | amend | gen | YLD kg/ha | Pht | NSPP | NPPP | AGB t/ha | SDW | RV | RDW | HSW | HSW |  |  |  |  |  |
| --- | --- | --- | --- | --- | --- | --- | --- | --- | --- | --- | --- | --- | --- | --- | --- | --- | --- | --- |
| 1 | C | B1 | 936.70 | 34.80 | 40.60 | 25.60 | 2.86 | 4.76 | 2.00 | 0.72 | 13.27 | 13.27 |  |  |  |  |  |
| 2 | C | B1 | 851.93 | 33.40 | 44.20 | 24.00 | 2.60 | 4.80 | 1.60 | 0.70 | 12.09 | 12.09 |  |  |  |  |  |
| 3 | C | B1 | 957.45 | 35.00 | 42.00 | 24.80 | 3.75 | 4.78 | 2.00 | 0.74 | 15.27 | 15.27 |  |  |  |  |  |
| 1 | C | B10 | 1040.48 | 44.60 | 43.00 | 20.00 | 4.00 | 4.54 | 2.00 | 0.60 | 17.38 | 17.38 |  | key |  |  |  |
| 2 | C | B10 | 1071.93 | 46.40 | 42.40 | 19.60 | 3.58 | 4.24 | 2.00 | 0.66 | 14.91 | 14.91 |  | NSPP= number of seed per plant | | | |
| 3 | C | B10 | 1176.95 | 43.60 | 45.20 | 21.80 | 3.12 | 4.38 | 2.00 | 0.62 | 13.40 | 13.40 |  |  |  |  |  |
| 1 | C | B11 | 1148.86 | 78.00 | 56.20 | 27.40 | 3.20 | 2.36 | 1.60 | 0.44 | 10.72 | 10.72 |  | NPPP= number of pod per plant | | | |
| 2 | C | B11 | 1069.87 | 68.60 | 55.00 | 25.40 | 3.00 | 2.21 | 1.40 | 0.42 | 10.29 | 10.29 |  |  |  |  |  |
| 3 | C | B11 | 990.89 | 73.20 | 56.40 | 27.00 | 3.49 | 2.30 | 2.00 | 0.44 | 11.34 | 11.34 |  | SWD= Shoot dry weight | | |  |
| 1 | C | B12 | 583.38 | 38.20 | 30.20 | 16.40 | 2.44 | 1.92 | 1.40 | 0.46 | 14.46 | 14.46 |  | NN=number of nodule | | per plant |  |
| 2 | C | B12 | 451.76 | 38.60 | 27.80 | 16.20 | 2.03 | 1.88 | 1.60 | 0.48 | 12.72 | 12.72 |  | RDW= root dry weight | | |  |
| 3 | C | B12 | 496.34 | 40.20 | 31.40 | 17.00 | 2.62 | 2.12 | 1.60 | 0.48 | 13.35 | 13.35 |  | Pht= plant height (cm) | | |  |
| 1 | C | B13 | 536.56 | 26.00 | 15.80 | 10.40 | 1.50 | 3.48 | 1.00 | 0.43 | 23.25 | 23.25 |  | Rv=root volume | |  |  |
| 2 | C | B13 | 487.70 | 27.40 | 13.80 | 9.40 | 1.22 | 3.98 | 1.60 | 0.45 | 16.70 | 16.70 |  | HSW= hundred seed weight | | |  |
| 3 | C | B13 | 560.38 | 25.20 | 13.40 | 9.20 | 1.57 | 3.62 | 1.40 | 0.40 | 14.66 | 14.66 |  |  |  |  |  |
| 1 | C | B14 | 899.16 | 29.20 | 36.00 | 17.40 | 2.32 | 4.14 | 2.00 | 0.60 | 11.08 | 11.08 |  |  |  |  |  |
| 2 | C | B14 | 707.02 | 30.40 | 35.20 | 18.80 | 1.53 | 3.96 | 1.00 | 0.54 | 8.10 | 8.10 |  |  |  |  |  |
| 3 | C | B14 | 859.18 | 29.20 | 39.40 | 17.80 | 2.97 | 3.98 | 2.00 | 0.60 | 13.47 | 13.47 |  |  |  |  |  |
| 1 | C | B15 | 1553.11 | 49.40 | 41.20 | 22.80 | 3.30 | 5.76 | 2.00 | 0.80 | 13.41 | 13.41 |  |  |  |  |  |
| 2 | C | B15 | 1416.66 | 52.40 | 40.20 | 22.60 | 4.43 | 6.08 | 2.00 | 0.80 | 13.84 | 13.84 |  |  |  |  |  |
| 3 | C | B15 | 1689.57 | 49.20 | 43.20 | 23.40 | 4.87 | 5.66 | 3.00 | 0.82 | 18.16 | 18.16 |  |  |  |  |  |
| 1 | C | B2 | 1093.13 | 48.20 | 35.60 | 19.20 | 3.30 | 4.24 | 3.00 | 0.73 | 15.10 | 15.10 |  |  |  |  |  |
| 2 | C | B2 | 956.93 | 45.60 | 37.80 | 19.20 | 3.97 | 3.72 | 2.00 | 0.74 | 15.71 | 15.71 |  |  |  |  |  |
| 3 | C | B2 | 1031.66 | 47.20 | 38.00 | 20.00 | 3.47 | 4.03 | 2.00 | 0.74 | 14.22 | 14.22 |  |  |  |  |  |
| 1 | C | B3 | 767.98 | 42.80 | 34.80 | 18.40 | 3.45 | 2.92 | 2.00 | 0.76 | 14.77 | 14.77 |  |  |  |  |  |
| 2 | C | B3 | 614.19 | 43.00 | 31.80 | 17.00 | 2.44 | 3.18 | 3.00 | 0.76 | 14.14 | 14.14 |  |  |  |  |  |
| 3 | C | B3 | 548.29 | 42.40 | 33.80 | 18.80 | 2.79 | 2.66 | 2.00 | 0.74 | 14.10 | 14.10 |  |  |  |  |  |
| 1 | C | B4 | 1020.74 | 44.20 | 44.60 | 21.00 | 4.06 | 5.58 | 3.00 | 0.90 | 17.56 | 17.56 |  |  |  |  |  |
| 2 | C | B4 | 1149.28 | 44.60 | 43.00 | 21.20 | 3.90 | 5.38 | 3.00 | 0.80 | 17.12 | 17.12 |  |  |  |  |  |
| 3 | C | B4 | 1194.02 | 43.80 | 46.40 | 21.80 | 3.92 | 5.40 | 2.00 | 0.92 | 16.48 | 16.48 |  |  |  |  |  |
| 1 | C | B5 | 651.87 | 42.00 | 36.60 | 19.00 | 2.96 | 2.48 | 2.00 | 0.66 | 14.18 | 14.18 |  |  |  |  |  |
| 2 | C | B5 | 623.91 | 42.00 | 36.00 | 18.00 | 2.34 | 2.32 | 1.00 | 0.60 | 13.48 | 13.48 |  |  |  |  |  |
| 3 | C | B5 | 636.85 | 40.60 | 33.20 | 19.80 | 2.90 | 2.34 | 2.00 | 0.68 | 15.46 | 15.46 |  |  |  |  |  |
| 1 | C | B6 | 1267.45 | 49.80 | 45.60 | 27.60 | 3.75 | 6.06 | 2.00 | 0.82 | 15.42 | 15.42 |  |  |  |  |  |
| 2 | C | B6 | 1375.02 | 48.20 | 50.80 | 28.80 | 4.24 | 5.98 | 3.00 | 0.86 | 16.55 | 16.55 |  |  |  |  |  |
| 3 | C | B6 | 1317.03 | 46.00 | 46.80 | 26.40 | 3.39 | 5.82 | 2.00 | 0.82 | 12.19 | 12.19 |  |  |  |  |  |
| 1 | C | B7 | 648.35 | 33.60 | 33.20 | 17.60 | 2.48 | 5.04 | 2.00 | 0.60 | 13.60 | 13.60 |  |  |  |  |  |
| 2 | C | B7 | 722.21 | 32.60 | 32.00 | 17.20 | 2.18 | 4.94 | 2.00 | 0.50 | 13.14 | 13.14 |  |  |  |  |  |
| 3 | C | B7 | 702.31 | 34.60 | 30.40 | 17.40 | 2.05 | 4.92 | 2.00 | 0.56 | 17.75 | 17.75 |  |  |  |  |  |
| 1 | C | B8 | 887.51 | 42.40 | 42.00 | 21.60 | 2.96 | 4.78 | 1.00 | 0.48 | 13.57 | 13.57 |  |  |  |  |  |
| 2 | C | B8 | 764.08 | 42.60 | 45.40 | 22.00 | 3.56 | 4.94 | 1.00 | 0.46 | 11.84 | 11.84 |  |  |  |  |  |
| 3 | C | B8 | 803.03 | 44.40 | 41.80 | 22.20 | 4.05 | 4.72 | 1.60 | 0.48 | 15.00 | 15.00 |  |  |  |  |  |
| 1 | C | B9 | 773.86 | 40.40 | 42.80 | 22.40 | 3.15 | 4.44 | 1.00 | 0.42 | 14.43 | 14.43 |  |  |  |  |  |
| 2 | C | B9 | 700.47 | 41.00 | 37.60 | 21.20 | 2.55 | 4.46 | 1.60 | 0.48 | 13.49 | 13.49 |  |  |  |  |  |
| 3 | C | B9 | 796.61 | 40.60 | 37.00 | 21.80 | 2.26 | 4.26 | 1.40 | 0.42 | 14.48 | 14.48 |  |  |  |  |  |
| 1 | L | B1 | 985.05 | 36.40 | 42.60 | 24.50 | 2.92 | 4.78 | 1.70 | 0.72 | 9.99 | 9.99 |  |  |  |  |  |
| 2 | L | B1 | 919.04 | 36.20 | 43.20 | 23.50 | 2.81 | 4.72 | 1.90 | 0.76 | 12.46 | 12.46 |  |  |  |  |  |
| 3 | L | B1 | 900.04 | 39.40 | 42.50 | 25.00 | 3.25 | 4.80 | 2.00 | 0.70 | 16.02 | 16.02 |  |  |  |  |  |
| 1 | L | B10 | 927.87 | 50.20 | 43.20 | 20.40 | 3.45 | 5.46 | 3.00 | 0.80 | 17.52 | 17.52 |  |  |  |  |  |
| 2 | L | B10 | 962.90 | 47.80 | 44.00 | 20.40 | 3.60 | 5.61 | 2.00 | 0.66 | 14.32 | 14.32 |  |  |  |  |  |
| 3 | L | B10 | 978.71 | 48.80 | 45.60 | 20.80 | 3.50 | 5.68 | 2.00 | 0.68 | 12.94 | 12.94 |  |  |  |  |  |
| 1 | L | B11 | 1797.25 | 85.20 | 92.80 | 47.00 | 6.45 | 6.44 | 2.00 | 0.74 | 11.01 | 11.01 |  |  |  |  |  |
| 2 | L | B11 | 1943.93 | 80.50 | 93.80 | 46.60 | 6.47 | 6.08 | 2.00 | 0.74 | 11.11 | 11.11 |  |  |  |  |  |
| 3 | L | B11 | 2090.61 | 85.50 | 90.40 | 47.60 | 8.15 | 6.44 | 3.00 | 0.76 | 12.02 | 12.02 |  |  |  |  |  |
| 1 | L | B12 | 660.51 | 44.20 | 31.60 | 17.20 | 2.41 | 3.80 | 2.00 | 0.68 | 12.80 | 12.80 |  |  |  |  |  |
| 2 | L | B12 | 592.66 | 42.50 | 30.00 | 18.00 | 2.95 | 3.48 | 3.00 | 0.90 | 11.40 | 11.40 |  |  |  |  |  |
| 3 | L | B12 | 603.68 | 47.80 | 34.60 | 18.60 | 2.38 | 3.52 | 3.00 | 0.82 | 15.14 | 15.14 |  |  |  |  |  |
| 1 | L | B13 | 667.79 | 33.60 | 23.80 | 13.80 | 2.21 | 6.56 | 2.00 | 0.62 | 16.42 | 16.42 |  |  |  |  |  |
| 2 | L | B13 | 660.34 | 31.20 | 22.00 | 13.00 | 1.57 | 6.64 | 3.00 | 0.58 | 16.66 | 16.66 |  |  |  |  |  |
| 3 | L | B13 | 720.33 | 32.00 | 20.60 | 13.20 | 1.82 | 6.26 | 2.00 | 0.58 | 18.78 | 18.78 |  |  |  |  |  |
| 1 | L | B14 | 775.26 | 32.40 | 41.20 | 21.60 | 2.52 | 5.26 | 2.00 | 0.64 | 11.09 | 11.09 |  |  |  |  |  |
| 2 | L | B14 | 772.49 | 29.60 | 37.40 | 21.20 | 2.56 | 5.26 | 2.00 | 0.64 | 9.61 | 9.61 |  |  |  |  |  |
| 3 | L | B14 | 769.70 | 34.00 | 40.40 | 21.20 | 2.23 | 5.02 | 2.00 | 0.60 | 9.83 | 9.83 |  |  |  |  |  |
| 1 | L | B15 | 1554.84 | 57.00 | 56.40 | 29.20 | 4.63 | 7.10 | 2.00 | 0.82 | 14.93 | 14.93 |  |  |  |  |  |
| 2 | L | B15 | 1590.63 | 54.00 | 61.60 | 29.40 | 5.26 | 6.90 | 2.00 | 0.78 | 12.96 | 12.96 |  |  |  |  |  |
| 3 | L | B15 | 1584.83 | 55.20 | 64.20 | 29.20 | 5.29 | 7.14 | 3.00 | 0.84 | 17.18 | 17.18 |  |  |  |  |  |
| 1 | L | B2 | 1362.73 | 56.60 | 42.80 | 21.60 | 4.22 | 5.22 | 2.00 | 0.80 | 13.49 | 13.49 |  |  |  |  |  |
| 2 | L | B2 | 1398.96 | 54.75 | 42.80 | 21.40 | 4.11 | 5.58 | 3.00 | 0.80 | 15.35 | 15.35 |  |  |  |  |  |
| 3 | L | B2 | 1223.18 | 52.80 | 45.40 | 21.60 | 4.38 | 5.40 | 3.00 | 0.78 | 14.22 | 14.22 |  |  |  |  |  |
| 1 | L | B3 | 892.13 | 49.60 | 39.60 | 23.00 | 3.41 | 4.46 | 2.00 | 0.90 | 14.42 | 14.42 |  |  |  |  |  |
| 2 | L | B3 | 889.52 | 48.00 | 40.00 | 22.60 | 3.33 | 4.19 | 2.00 | 0.94 | 13.60 | 13.60 |  |  |  |  |  |
| 3 | L | B3 | 1023.49 | 49.20 | 41.40 | 22.00 | 3.56 | 4.37 | 3.60 | 0.84 | 14.65 | 14.65 |  |  |  |  |  |
| 1 | L | B4 | 1198.40 | 46.40 | 45.40 | 21.00 | 3.95 | 6.15 | 4.00 | 1.05 | 15.09 | 15.09 |  |  |  |  |  |
| 2 | L | B4 | 1211.64 | 45.60 | 43.80 | 19.60 | 4.25 | 6.06 | 3.00 | 0.90 | 13.77 | 13.77 |  |  |  |  |  |
| 3 | L | B4 | 1233.33 | 46.80 | 45.50 | 20.20 | 3.83 | 6.28 | 4.00 | 0.86 | 18.34 | 18.34 |  |  |  |  |  |
| 1 | L | B5 | 647.59 | 45.60 | 38.40 | 21.00 | 2.33 | 2.97 | 2.20 | 0.72 | 12.91 | 12.91 |  |  |  |  |  |
| 2 | L | B5 | 641.33 | 45.40 | 40.20 | 21.20 | 3.65 | 2.81 | 3.00 | 0.68 | 12.70 | 12.70 |  |  |  |  |  |
| 3 | L | B5 | 670.60 | 46.60 | 38.60 | 21.60 | 3.21 | 3.06 | 2.00 | 0.72 | 11.37 | 11.37 |  |  |  |  |  |
| 1 | L | B6 | 1108.00 | 54.80 | 47.00 | 29.40 | 3.92 | 6.00 | 3.00 | 0.84 | 14.94 | 14.94 |  |  |  |  |  |
| 2 | L | B6 | 1190.10 | 52.60 | 45.80 | 31.60 | 3.83 | 5.90 | 2.00 | 0.86 | 13.46 | 13.46 |  |  |  |  |  |
| 3 | L | B6 | 1132.30 | 54.00 | 50.40 | 30.00 | 4.28 | 5.90 | 2.00 | 0.82 | 14.18 | 14.18 |  |  |  |  |  |
| 1 | L | B7 | 730.39 | 40.00 | 35.60 | 19.80 | 2.55 | 4.84 | 2.00 | 0.56 | 11.71 | 11.71 |  |  |  |  |  |
| 2 | L | B7 | 737.46 | 37.40 | 33.00 | 18.40 | 1.93 | 4.98 | 2.00 | 0.56 | 13.88 | 13.88 |  |  |  |  |  |
| 3 | L | B7 | 744.53 | 38.40 | 34.40 | 19.60 | 2.58 | 4.66 | 2.00 | 0.54 | 12.95 | 12.95 |  |  |  |  |  |
| 1 | L | B8 | 731.22 | 48.60 | 43.80 | 22.00 | 3.45 | 5.52 | 2.00 | 0.62 | 14.73 | 14.73 |  |  |  |  |  |
| 2 | L | B8 | 746.33 | 49.00 | 42.40 | 23.00 | 3.42 | 5.74 | 2.00 | 0.64 | 10.62 | 10.62 |  |  |  |  |  |
| 3 | L | B8 | 873.93 | 48.80 | 43.20 | 22.60 | 3.65 | 5.92 | 2.00 | 0.68 | 14.60 | 14.60 |  |  |  |  |  |
| 1 | L | B9 | 1008.84 | 44.40 | 41.40 | 23.00 | 2.80 | 7.52 | 1.40 | 0.66 | 12.58 | 12.58 |  |  |  |  |  |
| 2 | L | B9 | 1114.07 | 42.00 | 36.80 | 23.00 | 2.08 | 7.61 | 1.60 | 0.61 | 14.41 | 14.41 |  |  |  |  |  |
| 3 | L | B9 | 1105.82 | 42.80 | 38.60 | 22.80 | 3.49 | 7.60 | 2.00 | 0.64 | 12.54 | 12.54 |  |  |  |  |  |
| 1 | LP | B1 | 1032.58 | 45.80 | 43.40 | 25.20 | 3.22 | 7.28 | 3.00 | 0.72 | 13.19 | 13.19 |  |  |  |  |  |
| 2 | LP | B1 | 1072.18 | 44.80 | 42.20 | 27.40 | 2.71 | 7.44 | 2.00 | 0.73 | 12.13 | 12.13 |  |  |  |  |  |
| 3 | LP | B1 | 1075.37 | 42.80 | 42.40 | 26.00 | 3.35 | 7.16 | 2.00 | 0.81 | 15.84 | 15.84 |  |  |  |  |  |
| 1 | LP | B10 | 1460.56 | 61.80 | 51.80 | 28.40 | 4.82 | 7.48 | 3.00 | 1.04 | 18.25 | 18.25 |  |  |  |  |  |
| 2 | LP | B10 | 1395.24 | 61.00 | 50.20 | 28.20 | 4.32 | 7.81 | 3.00 | 0.98 | 15.14 | 15.14 |  |  |  |  |  |
| 3 | LP | B10 | 1430.37 | 62.00 | 50.00 | 29.00 | 5.35 | 7.44 | 3.60 | 1.03 | 14.72 | 14.72 |  |  |  |  |  |
| 1 | LP | B11 | 2073.15 | 83.20 | 99.20 | 49.00 | 6.85 | 6.54 | 3.70 | 0.92 | 10.13 | 10.13 |  |  |  |  |  |
| 2 | LP | B11 | 1989.82 | 82.40 | 94.00 | 47.50 | 6.95 | 6.69 | 3.60 | 0.94 | 10.09 | 10.09 |  |  |  |  |  |
| 3 | LP | B11 | 2086.94 | 84.00 | 95.40 | 48.80 | 7.35 | 6.64 | 3.90 | 0.91 | 11.56 | 11.56 |  |  |  |  |  |
| 1 | LP | B12 | 1083.73 | 52.80 | 40.40 | 23.00 | 3.17 | 5.23 | 3.00 | 0.95 | 11.60 | 11.60 |  |  |  |  |  |
| 2 | LP | B12 | 1162.34 | 55.60 | 43.40 | 22.80 | 3.60 | 5.54 | 3.18 | 1.17 | 13.35 | 13.35 |  |  |  |  |  |
| 3 | LP | B12 | 1276.82 | 54.60 | 40.80 | 22.80 | 4.38 | 5.52 | 3.00 | 1.04 | 15.12 | 15.12 |  |  |  |  |  |
| 1 | LP | B13 | 1985.90 | 43.60 | 40.40 | 26.80 | 4.21 | 8.54 | 3.60 | 0.86 | 24.92 | 24.92 |  |  |  |  |  |
| 2 | LP | B13 | 1914.39 | 41.80 | 43.60 | 25.20 | 4.27 | 8.70 | 3.50 | 0.84 | 19.43 | 19.43 |  |  |  |  |  |
| 3 | LP | B13 | 2044.62 | 38.60 | 41.60 | 25.80 | 4.22 | 8.68 | 3.10 | 0.90 | 21.80 | 21.80 |  |  |  |  |  |
| 1 | LP | B14 | 1462.75 | 40.60 | 58.80 | 28.20 | 3.60 | 8.30 | 2.00 | 0.82 | 13.23 | 13.23 |  |  |  |  |  |
| 2 | LP | B14 | 1454.34 | 40.60 | 62.40 | 28.80 | 4.60 | 8.70 | 2.00 | 0.86 | 12.93 | 12.93 |  |  |  |  |  |
| 3 | LP | B14 | 1532.47 | 39.00 | 58.20 | 28.60 | 3.99 | 8.56 | 2.00 | 0.86 | 13.94 | 13.94 |  |  |  |  |  |
| 1 | LP | B15 | 1989.42 | 63.40 | 59.20 | 34.00 | 5.27 | 9.96 | 4.00 | 1.06 | 20.05 | 20.05 |  |  |  |  |  |
| 2 | LP | B15 | 2077.10 | 62.60 | 57.00 | 33.00 | 6.43 | 10.04 | 3.40 | 1.08 | 17.69 | 17.69 |  |  |  |  |  |
| 3 | LP | B15 | 2075.04 | 62.00 | 60.40 | 35.00 | 6.20 | 9.84 | 3.20 | 1.04 | 15.20 | 15.20 |  |  |  |  |  |
| 1 | LP | B2 | 1577.22 | 60.80 | 63.00 | 32.60 | 5.67 | 8.30 | 4.00 | 1.14 | 15.96 | 15.96 |  |  |  |  |  |
| 2 | LP | B2 | 1762.52 | 59.60 | 61.00 | 30.40 | 6.96 | 8.50 | 4.00 | 1.30 | 18.08 | 18.08 |  |  |  |  |  |
| 3 | LP | B2 | 1583.97 | 58.60 | 60.00 | 31.40 | 6.11 | 8.10 | 4.00 | 1.32 | 14.11 | 14.11 |  |  |  |  |  |
| 1 | LP | B3 | 1346.00 | 53.20 | 48.40 | 28.40 | 4.26 | 5.56 | 3.00 | 1.31 | 14.97 | 14.97 |  |  |  |  |  |
| 2 | LP | B3 | 1439.75 | 53.40 | 46.60 | 27.40 | 4.03 | 5.76 | 3.00 | 1.12 | 14.46 | 14.46 |  |  |  |  |  |
| 3 | LP | B3 | 1439.36 | 55.20 | 50.20 | 28.20 | 5.07 | 5.56 | 4.00 | 1.12 | 15.80 | 15.80 |  |  |  |  |  |
| 1 | LP | B4 | 1714.00 | 53.60 | 43.60 | 24.00 | 3.67 | 8.15 | 4.00 | 1.29 | 15.98 | 15.98 |  |  |  |  |  |
| 2 | LP | B4 | 1974.47 | 52.00 | 46.40 | 23.60 | 4.41 | 8.24 | 4.00 | 1.30 | 15.49 | 15.49 |  |  |  |  |  |
| 3 | LP | B4 | 1809.41 | 55.00 | 45.80 | 24.80 | 5.27 | 8.10 | 4.00 | 1.18 | 18.31 | 18.31 |  |  |  |  |  |
| 1 | LP | B5 | 1109.05 | 54.80 | 48.60 | 28.60 | 4.35 | 5.18 | 4.00 | 1.11 | 13.68 | 13.68 |  |  |  |  |  |
| 2 | LP | B5 | 1200.49 | 52.00 | 45.20 | 28.00 | 4.12 | 4.85 | 4.00 | 1.07 | 11.70 | 11.70 |  |  |  |  |  |
| 3 | LP | B5 | 1336.93 | 54.80 | 47.80 | 30.40 | 4.59 | 5.04 | 3.00 | 1.12 | 16.25 | 16.25 |  |  |  |  |  |
| 1 | LP | B6 | 1294.99 | 57.80 | 69.80 | 38.80 | 5.66 | 7.42 | 4.00 | 1.20 | 14.58 | 14.58 |  |  |  |  |  |
| 2 | LP | B6 | 1321.85 | 57.80 | 66.20 | 37.40 | 5.85 | 7.64 | 3.00 | 1.18 | 13.31 | 13.31 |  |  |  |  |  |
| 3 | LP | B6 | 1348.71 | 57.20 | 69.60 | 37.80 | 6.75 | 7.50 | 4.00 | 1.20 | 13.91 | 13.91 |  |  |  |  |  |
| 1 | LP | B7 | 1883.06 | 50.60 | 69.00 | 36.80 | 5.89 | 9.26 | 2.00 | 0.91 | 16.40 | 16.40 |  |  |  |  |  |
| 2 | LP | B7 | 1765.60 | 52.40 | 70.60 | 36.60 | 6.64 | 9.06 | 2.60 | 0.92 | 14.63 | 14.63 |  |  |  |  |  |
| 3 | LP | B7 | 1842.44 | 52.80 | 73.20 | 35.00 | 6.00 | 9.34 | 3.00 | 0.90 | 17.94 | 17.94 |  |  |  |  |  |
| 1 | LP | B8 | 1068.18 | 54.40 | 53.20 | 34.80 | 4.25 | 7.52 | 2.40 | 0.74 | 14.87 | 14.87 |  |  |  |  |  |
| 2 | LP | B8 | 1125.68 | 53.60 | 56.40 | 34.60 | 4.80 | 7.76 | 3.00 | 0.72 | 13.48 | 13.48 |  |  |  |  |  |
| 3 | LP | B8 | 1175.25 | 55.00 | 55.00 | 36.40 | 5.00 | 7.40 | 3.00 | 0.72 | 16.06 | 16.06 |  |  |  |  |  |
| 1 | LP | B9 | 1076.11 | 50.40 | 48.00 | 25.00 | 3.15 | 7.59 | 2.00 | 0.60 | 14.58 | 14.58 |  |  |  |  |  |
| 2 | LP | B9 | 1132.47 | 48.80 | 46.80 | 25.00 | 2.87 | 7.12 | 1.60 | 0.58 | 13.03 | 13.03 |  |  |  |  |  |
| 3 | LP | B9 | 1154.59 | 50.40 | 45.60 | 25.00 | 3.39 | 7.34 | 1.60 | 0.56 | 13.53 | 13.53 |  |  |  |  |  |
| 1 | P | B1 | 907.53 | 40.80 | 44.40 | 26.60 | 3.11 | 6.15 | 2.00 | 0.66 | 16.39 | 16.39 |  |  |  |  |  |
| 2 | P | B1 | 828.16 | 40.60 | 41.60 | 25.00 | 2.64 | 5.88 | 2.00 | 0.74 | 14.71 | 14.71 |  |  |  |  |  |
| 3 | P | B1 | 898.52 | 38.00 | 40.40 | 26.60 | 3.38 | 6.22 | 2.00 | 0.64 | 14.10 | 14.10 |  |  |  |  |  |
| 1 | P | B10 | 1300.02 | 59.80 | 43.20 | 23.00 | 4.67 | 7.28 | 3.00 | 1.03 | 20.79 | 20.79 |  |  |  |  |  |
| 2 | P | B10 | 1497.99 | 60.80 | 42.00 | 22.20 | 4.29 | 7.37 | 3.00 | 0.91 | 16.30 | 16.30 |  |  |  |  |  |
| 3 | P | B10 | 1235.54 | 57.00 | 43.80 | 22.20 | 3.83 | 7.30 | 3.00 | 1.02 | 14.66 | 14.66 |  |  |  |  |  |
| 1 | P | B11 | 1539.90 | 83.60 | 76.40 | 33.60 | 5.98 | 6.16 | 4.00 | 0.90 | 10.16 | 10.16 |  |  |  |  |  |
| 2 | P | B11 | 1597.27 | 83.80 | 79.40 | 34.60 | 5.33 | 5.69 | 3.00 | 0.92 | 10.76 | 10.76 |  |  |  |  |  |
| 3 | P | B11 | 1466.23 | 85.20 | 75.80 | 33.00 | 4.24 | 5.90 | 3.00 | 0.90 | 10.50 | 10.50 |  |  |  |  |  |
| 1 | P | B12 | 933.90 | 49.20 | 40.00 | 22.00 | 3.15 | 4.00 | 3.00 | 1.03 | 15.02 | 15.02 |  |  |  |  |  |
| 2 | P | B12 | 929.32 | 50.00 | 37.00 | 22.00 | 3.76 | 4.21 | 3.00 | 1.02 | 11.62 | 11.62 |  |  |  |  |  |
| 3 | P | B12 | 1039.11 | 48.40 | 39.40 | 23.40 | 3.60 | 4.14 | 3.00 | 0.84 | 14.61 | 14.61 |  |  |  |  |  |
| 1 | P | B13 | 1613.69 | 39.00 | 40.00 | 23.20 | 4.60 | 6.92 | 4.00 | 0.90 | 26.02 | 26.02 |  |  |  |  |  |
| 2 | P | B13 | 1528.65 | 36.80 | 37.60 | 23.20 | 3.28 | 7.24 | 3.00 | 0.92 | 18.48 | 18.48 |  |  |  |  |  |
| 3 | P | B13 | 1515.66 | 39.00 | 40.20 | 23.40 | 4.82 | 7.02 | 3.00 | 0.84 | 21.54 | 21.54 |  |  |  |  |  |
| 1 | P | B14 | 1155.37 | 40.60 | 59.60 | 26.80 | 4.91 | 4.66 | 1.00 | 0.55 | 15.44 | 15.44 |  |  |  |  |  |
| 2 | P | B14 | 1154.99 | 36.80 | 55.60 | 25.20 | 3.19 | 4.42 | 1.40 | 0.55 | 11.51 | 11.51 |  |  |  |  |  |
| 3 | P | B14 | 1209.60 | 37.80 | 55.80 | 25.80 | 3.61 | 4.56 | 1.60 | 0.54 | 11.29 | 11.29 |  |  |  |  |  |
| 1 | P | B15 | 2165.70 | 56.80 | 56.20 | 26.60 | 5.86 | 10.18 | 4.00 | 1.11 | 16.18 | 16.18 |  |  |  |  |  |
| 2 | P | B15 | 2097.08 | 59.40 | 52.80 | 26.00 | 5.25 | 9.70 | 3.00 | 1.01 | 15.77 | 15.77 |  |  |  |  |  |
| 3 | P | B15 | 2097.29 | 58.20 | 56.40 | 27.40 | 6.60 | 9.81 | 4.00 | 1.07 | 17.99 | 17.99 |  |  |  |  |  |
| 1 | P | B2 | 1476.91 | 55.40 | 55.40 | 26.20 | 5.07 | 7.32 | 4.00 | 1.26 | 14.42 | 14.42 |  |  |  |  |  |
| 2 | P | B2 | 1564.55 | 57.40 | 53.00 | 26.60 | 5.30 | 7.20 | 4.00 | 1.30 | 14.27 | 14.27 |  |  |  |  |  |
| 3 | P | B2 | 1385.93 | 57.40 | 56.00 | 27.20 | 6.42 | 7.04 | 4.00 | 1.28 | 15.75 | 15.75 |  |  |  |  |  |
| 1 | P | B3 | 1073.39 | 46.60 | 33.80 | 18.80 | 2.61 | 3.66 | 2.00 | 1.04 | 12.92 | 12.92 |  |  |  |  |  |
| 2 | P | B3 | 817.64 | 45.80 | 36.20 | 19.40 | 3.12 | 3.41 | 3.00 | 0.96 | 13.20 | 13.20 |  |  |  |  |  |
| 3 | P | B3 | 804.27 | 45.60 | 35.40 | 19.60 | 3.44 | 3.82 | 3.00 | 0.98 | 13.67 | 13.67 |  |  |  |  |  |
| 1 | P | B4 | 1553.35 | 54.20 | 45.20 | 24.00 | 4.43 | 8.46 | 4.00 | 1.29 | 15.41 | 15.41 |  |  |  |  |  |
| 2 | P | B4 | 1529.06 | 51.00 | 47.40 | 24.60 | 4.63 | 7.97 | 4.00 | 1.24 | 13.57 | 13.57 |  |  |  |  |  |
| 3 | P | B4 | 1765.28 | 52.20 | 47.80 | 23.40 | 4.57 | 8.00 | 4.00 | 1.26 | 16.58 | 16.58 |  |  |  |  |  |
| 1 | P | B5 | 1160.32 | 49.40 | 50.20 | 27.00 | 4.78 | 4.43 | 3.00 | 1.07 | 15.82 | 15.82 |  |  |  |  |  |
| 2 | P | B5 | 1219.01 | 50.80 | 46.40 | 26.80 | 4.10 | 4.74 | 3.00 | 1.08 | 14.24 | 14.24 |  |  |  |  |  |
| 3 | P | B5 | 1012.00 | 49.00 | 46.60 | 26.60 | 4.03 | 4.62 | 3.60 | 1.10 | 18.26 | 18.26 |  |  |  |  |  |
| 1 | P | B6 | 1546.49 | 60.80 | 71.00 | 38.80 | 6.44 | 7.46 | 3.00 | 1.17 | 16.50 | 16.50 |  |  |  |  |  |
| 2 | P | B6 | 1535.47 | 58.80 | 70.00 | 38.00 | 6.84 | 7.10 | 3.80 | 1.22 | 11.10 | 11.10 |  |  |  |  |  |
| 3 | P | B6 | 1338.02 | 58.60 | 67.20 | 37.60 | 5.73 | 7.62 | 3.00 | 1.18 | 15.32 | 15.32 |  |  |  |  |  |
| 1 | P | B7 | 1342.24 | 41.60 | 43.00 | 24.60 | 4.59 | 6.22 | 2.00 | 0.84 | 20.06 | 20.06 |  |  |  |  |  |
| 2 | P | B7 | 1295.94 | 41.80 | 47.80 | 25.60 | 3.41 | 6.07 | 2.00 | 0.83 | 15.44 | 15.44 |  |  |  |  |  |
| 3 | P | B7 | 1224.99 | 42.80 | 45.00 | 24.40 | 3.69 | 6.20 | 2.00 | 0.90 | 16.70 | 16.70 |  |  |  |  |  |
| 1 | P | B8 | 1209.65 | 47.20 | 48.80 | 27.00 | 4.39 | 6.72 | 2.40 | 0.71 | 15.95 | 15.95 |  |  |  |  |  |
| 2 | P | B8 | 1146.89 | 48.00 | 50.20 | 25.20 | 3.50 | 6.47 | 2.80 | 0.69 | 12.76 | 12.76 |  |  |  |  |  |
| 3 | P | B8 | 1185.37 | 49.00 | 45.20 | 26.40 | 3.46 | 6.92 | 2.00 | 0.76 | 13.84 | 13.84 |  |  |  |  |  |
| 1 | P | B9 | 971.64 | 46.80 | 48.00 | 23.20 | 3.62 | 6.21 | 1.00 | 0.61 | 16.51 | 16.51 |  |  |  |  |  |
| 2 | P | B9 | 810.65 | 45.00 | 44.40 | 23.20 | 3.17 | 5.86 | 1.00 | 0.54 | 14.61 | 14.61 |  |  |  |  |  |
| 3 | P | B9 | 936.00 | 44.00 | 45.60 | 23.00 | 3.29 | 6.34 | 1.00 | 0.54 | 13.31 | 13.31 |  |  |  |  |  |

| key |
| --- |
| NSPP= number of seed per plant |
| NPPP= number of pod per plant |
| SWD= Shoot dry weight |
| NN=number of nodule per plant |
| RDW= root dry weight |
| Pht= plant height (cm) |
| Rv=root volume |
| HSW= hundred seed weight |

**C= control (without lime),**

Amend= amendment,

Gen= genotypes

B1……B15 = genotype code
