# Supplementary material for: Tolerance to soil acidity of soybean (Glycine max L.) genotypes under field conditions Southwestern Ethiopia
Source: PLoS One. 2022 Sep 15;17(9):e0272924. doi: 10.1371/journal.pone.0272924 (PMC9477287; doi:10.1371/journal.pone.0272924)
Supplement: Attachment — Submitted filename: PONE-D-20-38977_reviewer.pdf [file pone.0272924.s002.pdf]

PLOS ONE

Tolerance to soil acidity of soybean (Glycine max L.) genotypes under field conditions at Southwestern Ethiopia

--Manuscript Draft--

**Manuscript Number:**	PONE-D-20-38977
**Article Type:**	Research Article
**Full Title:**	Tolerance to soil acidity of soybean (Glycine max L.) genotypes under field conditions at Southwestern Ethiopia
**Short Title:**	Tolerance to Soil acidity of Soybean (Glycine max L.) Genotypes
**Corresponding Author:**	Tolossa Ameyu Bedassa, Msc<br>Ethiopian Institute of Agricultural Research<br>Jimma, Oromia, Ethiopia ETHIOPIA
**Keywords:**	soybean; Soil acidity; Genotypes; Lime
**Abstract:**	Soil acidity limits the productive potential of crops, mainly because of low availability of basic cations, and excess of hydrogen and aluminium in exchangeable forms. This study was conducted to assess the response of soybean genotypes to soil acidity for some of the important yield and yield related traits, and root and growth parameters on contrasting i.e., lime treated and untreated acidic soil of Mettu, Southwestern Ethiopia. The study revealed that genotype x liming interactions were significant (p<0.01) for all growth, root and nodulation characteristics and yield and yield components, except for hundred seed weight and root volume where only the main effects of genotypes and liming were significant. The study also revealed considerable difference among the genotypes tested based on the root, growth, nodulation and yield components measured for soil acidity tolerance. Though some genotypes showed higher performance for root and other growth parameters, and yield components under unlimed soils; however, gave higher yield and yield components, when grown on lime treated than lime untreated soil with average yield reduction of 13.7%, due to soil acidity effect. The maximum grain yield of (1943.93 kg ha $^{-1}$) was obtained under lime treated acid soil from PI567046A genotype; while the lowest (510.49 kg ha $^{-1}$) were recorded from SCS-1genotype under the lime untreated acid soil. Genotype, BRS268 showed higher yield(1319.83 kg ha $^{-1}$) under lime untreated acid soil than lime treated acid soil(1143.47 kg ha $^{-1}$) and showed less reduction percentage for number of nodule, root weight and number of seeds per plant; while PI567046A showed high reduction percentage for yield, biomass, number of pod and seed per plant. In general, high difference was observed among the soybean genotypes for soil acidity tolerance, which might be further exploited by breeders for the genetic improvement of soybean.
**Order of Authors:**	Tolossa Ameyu Bedassa, Msc
	Abush Tesfaye Abebe
	Alemayehu Regassa Tolessa
**Additional Information:**	
**Question**	**Response**
**Financial Disclosure**<br><br>Enter a financial disclosure statement that describes the sources of funding for the work included in this submission. Review the submission guidelines for detailed requirements. View published research articles from *PLOS ONE* for specific examples.	NO, The funders had no role in study design, data collection and analysis, decision to publish, or preparation of the manuscript.

Powered by Editorial Manager® and ProduXion Manager® from Aries Systems Corporation

This statement is required for submission and **will appear in the published article** if the submission is accepted. Please make sure it is accurate.<br><br>**Unfunded studies**<br>Enter: *The author(s) received no specific funding for this work.*<br><br>**Funded studies**<br>Enter a statement with the following details:<br>• Initials of the authors who received each award<br>• Grant numbers awarded to each author<br>• The full name of each funder<br>• URL of each funder website<br>• Did the sponsors or funders play any role in the study design, data collection and analysis, decision to publish, or preparation of the manuscript?<br>• **NO** - Include this sentence at the end of your statement: *The funders had no role in study design, data collection and analysis, decision to publish, or preparation of the manuscript.*<br>• **YES** - Specify the role(s) played.<br><br>* typeset	
**Competing Interests**<br><br>Use the instructions below to enter a competing interest statement for this submission. On behalf of all authors, disclose any competing interests that could be perceived to bias this work—acknowledging all financial support and any other relevant financial or non-financial competing interests.<br><br>This statement **will appear in the published article** if the submission is accepted. Please make sure it is accurate. View published research articles from *PLOS ONE* for specific examples.	The authors have declared that no competing interests exist.

**NO authors have competing interests**<br><br>Enter: *The authors have declared that no competing interests exist.*<br><br>**Authors with competing interests**<br><br>Enter competing interest details beginning with this statement:<br><br>*I have read the journal's policy and the authors of this manuscript have the following competing interests: [insert competing interests here]*<br><br>* typeset	
**Ethics Statement**<br><br>Enter an ethics statement for this submission. This statement is required if the study involved:<br><br>• Human participants<br>• Human specimens or tissue<br>• Vertebrate animals or cephalopods<br>• Vertebrate embryos or tissues<br>• Field research<br><br>Write "N/A" if the submission does not require an ethics statement.<br><br>General guidance is provided below. Consult the submission guidelines for detailed instructions. **Make sure that all information entered here is included in the Methods section of the manuscript.**	Ethiopian inistitute of agricultural research

Powered by Editorial Manager® and ProduXion Manager® from Aries Systems Corporation

**Format for specific study types**<br><br>**Human Subject Research (involving human participants and/or tissue)**<br>• Give the name of the institutional review board or ethics committee that approved the study<br>• Include the approval number and/or a statement indicating approval of this research<br>• Indicate the form of consent obtained (written/oral) or the reason that consent was not obtained (e.g. the data were analyzed anonymously)<br><br>**Animal Research (involving vertebrate animals, embryos or tissues)**<br>• Provide the name of the Institutional Animal Care and Use Committee (IACUC) or other relevant ethics board that reviewed the study protocol, and indicate whether they approved this research or granted a formal waiver of ethical approval<br>• Include an approval number if one was obtained<br>• If the study involved *non-human primates*, add *additional details* about animal welfare and steps taken to ameliorate suffering<br>• If anesthesia, euthanasia, or any kind of animal sacrifice is part of the study, include briefly which substances and/or methods were applied<br><br>**Field Research**<br><br>Include the following details if this study involves the collection of plant, animal, or other materials from a natural setting:<br>• Field permit number<br>• Name of the institution or relevant body that granted permission	
**Data Availability**<br><br>Authors are required to make all data underlying the findings described fully available, without restriction, and from the time of publication. PLOS allows rare exceptions to address legal and ethical concerns. See the PLOS Data Policy and FAQ for detailed information.	No - some restrictions will apply

Powered by Editorial Manager® and ProduXion Manager® from Aries Systems Corporation

A Data Availability Statement describing where the data can be found is required at submission. Your answers to this question constitute the Data Availability Statement and **will be published in the article**, if accepted.<br><br>**Important:** Stating 'data available on request from the author' is not sufficient. If your data are only available upon request, select 'No' for the first question and explain your exceptional situation in the text box.<br><br>Do the authors confirm that all data underlying the findings described in their manuscript are fully available without restriction?	
**Describe where the data may be found in full sentences. If you are copying our sample text, replace any instances of XXX with the appropriate details.**<br><br>• If the data are **held or will be held in a public repository**, include URLs, accession numbers or DOIs. If this information will only be available after acceptance, indicate this by ticking the box below. For example: *All XXX files are available from the XXX database (accession number(s) XXX, XXX.).*<br>• If the data are all contained **within the manuscript and/or Supporting Information files**, enter the following: *All relevant data are within the manuscript and its Supporting Information files.*<br>• If neither of these applies but you are able to provide **details of access elsewhere**, with or without limitations, please do so. For example:<br><br>*Data cannot be shared publicly because of [XXX]. Data are available from the XXX Institutional Data Access / Ethics Committee (contact via XXX) for researchers who meet the criteria for access to confidential data.*<br><br>*The data underlying the results presented in the study are available from (include the name of the third party*	All relevant data are within the manuscript and its Supporting Information files.

and contact information or URL).<br>• This text is appropriate if the data are owned by a third party and authors do not have permission to share the data.<br><br>* typeset	
Additional data availability information:	Tick here if the URLs/accession numbers/DOIs will be available only after acceptance of the manuscript for publication so that we can ensure their inclusion before publication.

Powered by Editorial Manager® and ProduXion Manager® from Aries Systems Corporation

manuscript Click here to access/download;Manuscript;Acid tolerant paper.doc

Tolerance to soil acidity of soybean (*Glycine max L.)* genotypes under field conditions at Southwestern Ethiopia

Tolossa Ameyu[1], Abush Tesfaye[2] and Alemayehu Regassa[3]
[1,2]Ethiopian Institute of Agricultural Research; Jimma Research Center P.O Box 192, Jimma, Ethiopia.
2. International Institute of Tropical Agriculture, PMB 5320, Oyo road 200001, Ibadan, Nigeria.
[3]Department of Natural Resource Management, Jimma University, Post Box 378, Jimma, Ethiopia

Abstract

Soil acidity limits the productive potential of crops, mainly because of low availability of basic cations, and excess of hydrogen and aluminium in exchangeable forms. This study was conducted to assess the response of soybean genotypes to soil acidity for some of the important yield and yield related traits, and root and growth parameters on contrasting i.e., lime treated and untreated acidic soil of Mettu, Southwestern Ethiopia. The study revealed that genotype x liming interactions were significant ($p<0.01$) for all growth, root and nodulation characteristics and yield and yield components, except for hundred seed weight and root volume where only the main effects of genotypes and liming were significant. The study also revealed considerable difference among the genotypes tested based on the root, growth, nodulation and yield components measured for soil acidity tolerance. Though some genotypes showed higher performance for root and other growth parameters, and yield components under unlimed soils; however, gave higher yield and yield components, when grown on lime treated than lime untreated soil with average yield reduction of 13.7%, due to soil acidity effect. The maximum grain yield of (1943.93 kg ha^{-1}) was obtained under lime treated acid soil from PI567046A genotype; while the lowest (510.49 kg ha^{-1}) were recorded from SCS-1genotype under the lime untreated acid soil. Genotype, BRS268 showed higher yield(1319.83 kg ha^{-1}) under lime untreated acid soil than lime treated acid soil(1143.47 kg ha^{-1}) and showed less reduction percentage for number of nodule, root weight and number of seeds per plant; while PI567046A showed high reduction percentage for yield, biomass, number of pod and seed per plant. In general, high difference was observed among the soybean genotypes for soil acidity tolerance, which might be further exploited by breeders for the genetic improvement of soybean.

Key words: *Soybean; Soil acidity; Genotypes; Lime*

Introduction

Soil acidity is one of the major problematic soils that has profound effect on the productive potential of crops, such as soybean, because of low availability of basic cations, and excess and toxic levels of hydrogen and aluminum in exchangeable forms (Fageria and Baligar, 2008). Leaching of exchangeable bases, uptake of basic cation by plants, decomposition of organic materials, application of commercial fertilizers, the parent materials the soil is formed from and other farming practices has high contribution to the occurrence and aggravation of soil acidity (Brady and Weil, 2002). Soil acidity is often an insidious soil degradation process, developing slowly; although indicators, such as falling yields, leaf discolorations in susceptible plants, and lack of response to fertilizers might indicate that soil pH is declining to critical levels.

Theoretically, soil acidity is quantified based on H^{+} and Al^{3+} concentrations of the soils (Moody and Cong, 2008). In terms affecting crop production, soil acidity is a complex of numerous factors involving nutrient deficiencies and toxicities, low activities of beneficial microorganisms, and reduced root growth, which limits absorption of nutrients and water (Fageria and Baligar, 2008). However, Al^{3+} toxicity is one of the major limiting factors of crop production on acid soils through inhibiting root cell division and elongation, thereby, reducing water and nutrient uptake (Wang *et al*., 2006), poor nodulation or mycorrhizal infections (Delhaize *et al*., 2007), consequently, leading to poor plant growth and yield of crops.

Acid soil is mostly distributed in developing countries, where there is high population growth, and food demand is ever increasing. Globally, soil acidity comprised of 50% of the world's poten tially arable land, and hence, it is a very important factor that limits the production and productivity of crops (Uexkull and Muter, 1995). According to Mesfin (2007), about 40.9 % of the total land in Ethiopia is affected by soil acidity, of which, vast areas of land in the Western, Southern, South-western, and North-western and in the central highlands of the country that are known for receiving high rainfall are reported affected by soil acidity.

Soybean production and productivity has been growing rapidly in Ethiopia, in the past decade, and according to the report of CSA (2016), 130,022.00 private peasant holdings that cultivated about 36,635.79 hectares of land produced 812, 34.659 ton of soybean. The national average productivity of the crop, therefore, is 2.22 t/ha; while its productivity in the study area is 1.3

ton/ha, which is by far below the national average productivity of the crop (CSA, 2016/17); while the potential productivity in the research plots may reach up to 3.5 t/ha (Abush *et al*., 2017). The reduction of soybean yield at the study area is due to soil acidity which involving nutrient deficiencies and toxicities, low activities of beneficial micro organisms, and reduced plant root growth, which limits absorption of nutrients and water (Fageria and Baligar, 2008). However, among nutrient toxicity, alumunium toxicity is one of the major limiting factors for crop production on acid soils by inhibiting root cell division and elongation, thereby, reducing water and nutrient uptake (Wang *et al*., 2006), poor nodulation or mycorrhizal infections (Delhaize *et al*., 2007), consequently leading to poor plant growth and yield of crops.

Lime and fertilizer management practices are primary importance for proper management of soil acidity. Nevertheless, for economic reasons, it is often not practicable for resource-poor farmers to apply high rates of lime (Uguru *et al*., 2012; Nyoki and Ndakidemi, 2014). However, previous studies revealed the existence of sufficient genotypic variability of bean germplasm for acid tolerance (Rao, 2001; Rangel *et al*., 2005; Manrique *et al*., 2006). Hence, development of soybean genotypes that have better tolerance to soil acidity, and perform relatively better than others on acidic soils, is economically feasible option that might serve as an alternative or supplement to liming, and other acid soil amendment practices. Hence, the identification and use of soybean genotypes that are tolerant to acid soil conditions of Southwestern Ethiopia is a very useful approach to ensure economic stability to many subsistence farmers, who cannot afford the application of liming materials. Therefore, the objective of the study was to assess the differential responses of soybean genotypes to soil acidity for yield and yield related traits, and root and growth parameters.

Materials and Methods

Description of the Study Site

The study was carried out at Mettu Agricultural Research Sub Center of the Jimma Agricultural Research Center, under the Ethiopian Institute of Agricultural Research (EIAR), during the 2017/18 main cropping season. The sub-center is located at 600 km away from Addis Ababa in Iluabbabora Zone of the Oromia Regional State. Geographically, it is located at latitude 8°19' 0" N longitude 35°35' 0"E at an altitude of 1550 m.a.s.l. Agro-climatically, it has been characterized as Tepid to cool, humid, mid-highlands with annual rainfall of 1835 mm/annum. The mean annual temperature of the experimental station ranges from 12 to 27 ^{0}C. The predominant soil type of the area is Nitisol, which is dark red brown, and characterized by very strong to moderate acidity, and low soil P, specifically around experimental sites with pH of 4.5, and phosphorus level of 1.16 ppm, and exchangeable acidity of 2.48 meq/100g of soil (Abush *et al.*, 2017). Physical and some chemical properties of the soil in the study area before sowing and after harve sting is presented in the table below.

Table1: Physicochemical properties of the experimental soil prior to sowing and after harvesting

Parameters	Before sowing	After harvesting	
Particle size distribution		Limed	Unlimed
Clay (%)	49.00		
Sand (%)	38.00		
Silt (%)	13.00		
Textural class	Clayey		
pH (H_2O)	4.400	4.73	4.48
Exchangeable acidity (cmol(+)/kg)	2.720	1.52	2.41
Exchangeable Al (cmol(+)/kg)	1.460	0.93	1.38
Organic carbon (%)	2.210	2.45	2.22
CEC (cmol (+) kg^{-1})	18.75	21.04	18.89
Total N (%)	0.210	0.24	0.22
Available P(BrayII)(mg kg^{-1})	2.950	4.39	2.98
Exchangeable K (cmol (+) kg^{-1})	0.330	0.67	0.40
Exchangeable Ca (cmol(+) kg^{-1})	3.550	5.39	3.81
Exchangeable Mg (cmol(+) kg^{-1})	1.380	1.59	1.40

Soil Sampling and Analysis

Prior to the field experimentation both undisturbed and disturbed samples were collected. Three undisturbed soil samples were taken by core sampler. Fresh weight and an oven dry weight at 105°C, and used to determine bulk density (Baruah *et al*., 1997). Five random disturbed soil samples (0-15cm depth) were collected diagonally and composite soil sample was made. The composite sample was used for soil physiochemical analysis, and for the determination of lime requirement of the soil. The disturbed soil samples were air dried and sieved to pass through 2 mm sieve, and placed in a labeled plastic bag. Then, the samples were transported to Jimma Agricultural Research Center soil laboratory for analysis, and the soil samples were analyzed for particle size distribution (soil texture), which was done by Bouyoucos hydrometer method as described by Bouyoucos (1962) that are among the physical soil parameters; while soil exchangeable acidity, exchangeable bases, soil pH, organic carbon (OC), total nitrogen (TN), available phosphorus and cation exchange capacity (CEC) for soil chemical analysis were selected.

After harvesting, the soil samples were collected from limed and un-limed soils at the depth of 0-15 cm, separately, for selected soil chemical analysis, and then the soil samples were air dried, sieved to pass through 2 mm sieve, placed in a labeled plastic bags and submitted to the soil laboratory for soil chemical properties analysis. Organic matter was determined using wet oxidation. Total N was determined by Kjeldahl method, as described by Black (1965). Cation exchange capacity (CEC) and exchangeable bases (Ca, Mg, K and Na) were determined after extracting the soil samples by ammonium acetate (1NH4OAc) at pH 7.0. Exchangeable Ca and Mg in the extracts were analyzed using Atomic Absorption Spectrophotom eter (AAS), and Na and K were analyzed by flame photometer as described by Chapman (1965) and Rowell (1994). Available soil P was determined using Bray-II method, as described by Bray and Kurtz (1945). The soil pH was determined in soil water suspension of 1:2.5 (soil: water ratio) using pH meter, as described by Van Reeuwijk (1992). Exchangeable acidity was determined by saturating the soil samples with potassium chloride solution and titrates with sodium hydroxide as described by Mclean (1965). From the same extractant, exchangeable Al in the soil was titrated with a standard solution of 0.02M HCl.

Determination of Lime Requirements

The amounts of lime applied was determined based on the exchangeable acidity, mass per 0.15m furrow slice and bulk density of the soil (Shoemaker et al., 1961; Van Lierop, 1983), considering the amount of lime needed to neutralize the acid content (Al + H) of the soil up to the permissible acid saturation level for soybean growth.

$$\frac{LR, \frac{CaCo3kg}{ha} = Cmol\frac{EA}{Kg}\, of\ soil * 0.15m * 10m^2 * BD\left(\frac{g}{cm^3}\right) * 1000 * crop\ factor}{2000}$$

Where: BD = bulk density, EA = exchangeable acidity (exch. H^+ + Al^{3+}), LR = lime requirement, 0.15m = plough depth/depth of lime incorporation.

Planting Material, Treatments, Experimental Design and Procedures

The treatments was comprised of two factors namely; two soil amendments (control or no lime and limed) and fifteen different soybean genotypes (Table 2). The treatments were laid out in split plot design with three replications, were soil amendments (lime and unlimed) applied as mai n plot treatments and soybean genotypes were applied to sub plot treatments. The different soybe an genotypes for the trial were identified from previous advanced Multi-Location Yield Trials, including previous soil acidity tolerance screening trials. The lime requirement (LR) of the soil for the plots was determined based on exchangeable acidity (EA) or acid saturation of the experimental soil. The lime rate was, therefore, 3457.8 kg/ha based on exchangeable acidity of the soil. Calcium carbonate (CaCO3) was used as the source of lime and the whole doses of lime were broadcasted on limed plots manually, uniformly and mixed in the top 15 cm soil layer, a month before sowing. Reduction percentage for grain yield, root, growth and nodulation parameter was calculated as the ratio in lime treated to lime untreated soil, which also showed higher differences among the tested genotypes. Soybean seeds were planted on June 20, 2017 as per the recommended soybean planting period. Two seeds were sown in rows per hill to maintain between plants and rows spacings of 5 and 60 cm, respectively, and then thinned to one plant per hill after seedling establishment. The size of each plot was 2.4 m x 4 m (9.6 m^2) and the spacing between replication and plots were 1.5 and 1 m, respectively. All the recommended cultural practices were used for the management of the experimental crop.

Table 2: Soybean Genotypes Used for the Experiment

Genotypes	Back ground information and Source
JM-DAV/PR 142-15-SA	Inbreed line from local crosses
JM-PR142/H3-15-SB	Inbreed line from local crosses
JM-CLK/CRFD-15-SA	Inbreed line from local crosses
JM-ALM/PR142-15-SC	Inbreed line from local crosses
JM-ALM/H3-15-SC-1	Inbreed line from local crosses
BRS 268	Introduced from Brazil
JM-HAR/DAV-15-SA	Inbreed line from local crosses
JM-CLK/G99-15-SC	Inbreed line from local crosses
JM-CLK/G99-15-SB	Inbreed line from local crosses
JM-H3/SCS-15-SG	Inbreed line from local crosses
Pl 567046A	Introduced from USA
SCS-1	Pipe line from Pawe agricultural research
Pl 423958	Introduced from USA
H-7	Pipe line from Mozambique ARI
HAWASSA- 04	Released variety from Hawassa

Data Collection and Measurements

Plant height was recorded on five randomly selected plants from the central two rows of each plot at flowering, and shoot dry weight was taken on the shoots of randomly selected five plants dried in an oven at 70 ^{O}c for 48 hrs to a constant weight. Nodule and root traits were measured by carefully uprooting five randomly selected plants along with the soil from each plot, and washing the root gently, not to damage the fibrous roots and not to lose the nodules. Root volume was measured for the root samples on randomly selected five plants that were carefully immersed in 1000 ml capacity plastic measuring cylinder, which is filled up with 700ml water, and the volume of water displaced by the root sample was recorded as root volume per plant; while root dry weight was measured on the roots of five plants that were dried at 70 ^{O}c for 48 hrs in an oven to a constant weight, and then weighted. Total number of nodule was taken as the count of all the nodules formed by the roots of five plants counted at the stage, where 50% of the plants flowered, and averaged as number of nodules per plant.

Number of pods and seeds per plant were counted from five randomly selected plants; while hundred seeds weight (g) was counted from randomly sampled seeds from harvested and bulked seeds, and weighed (g). Grain yield (g/plot) was measured from the harvestable middle two rows using a sensitive balance, and converted to kg ha^{-1} adjusting the seed moisture content at 10% seed moisture level (standard seed moisture content for pulse crops). The total above ground dry biomass weight was measured by taking the weight in kg measurement of the above ground

(from the surface of the soil up to the tip of the shoot) biomass of 10 randomly selected plants per plot at physiological maturity by drying the biomass in oven at 70oc for 24 hrs. Finally, the biomass yield of the selected 10 plants was converted to per ton per hectare.

Statistical Analysis

The data was subjected to analysis of variance (ANOVA) using Statistical Analysis System (SAS) software version 9.3 (SAS Institute, 2012) using proc GLM procedure. The difference between treatment means was separated using LSD 5% value. Correlation analysis between the traits was carried out to determine the magnitude and degree of their associations.

Results and Discussion

Effect of Soil Acidity on Yield and Yield Components of Soybean Genotypes

There were highly significant (P<0.001) differences among genotypes and amendment for number pods per plant, number of seed per plant, grain yield, hundred seed weight and above ground biomass in both limed and unlimed soil regimes (Table 3). The interaction of limin g*genotypes was also highly significant(p≤0.01) for all yield and yield components, except for hundred seed weight only the main effect of genotypes were significant. Low pH affects plant growth and development that were indicated by the response of its characters. The response of the observed characters in acid soil toxicity varied. Grain yield, number of pod per plant, number of seed per plant, hundred seed weight and above ground biomass in unlimed soil with low pH was lower than in limed soil or same with that in limed (Table 4). Even, grain yield of BRS268 genotype under unlimed acid soil were higher than in limed acid soil.

On average, the genotypes gave higher yield and yield components in lime treated soil (Table 4). PI567046A material gave higher grain yield, above ground biomass, number of pods and seeds per plant in lime treated acid soil; while genotype SCS-1 gave the lowest yield and genotype PI423958 gave the lowest number of pod, seed and above ground biomass in lime untreated acid soil (Table 4). Under lime untreated soil condition, the maximum grain yield and above ground biomass was obtained from variety HAWASSA-04, and genotypes BRS268 and PI567046A gave highest number of pods and seeds per plant respectively. Under unlimed acid soil condition, the highest increasing percentage for yield and above ground biomass, was shown

by BRS268 and JM-DAV/PR142-15-SA respectively, while the highest decreasing percentage was shown by PI567046A for both yield and above ground biomass.

The yield increments with lime application might be due to the probability of obtaining the available P from decomposed OM by microorganisms, when the pH value of the soil improved due to liming, which might have resulted in increased grain yield. Liming also improved the ability of the plant to absorb P, when Al toxicity has been eliminated, and enhanced the vegetative growth of soybean genotypes, which resulted in increased dry biomass yield. In line with this result, Temasgen *et al*. (2017) also reported that the highest barley grain yield was obtained under the application of 2.2 t/ha lime than unlimed acid soil. The genotypes responded to the applied lime for number of pod and seed, which might be due to lime enhanced vegetative growth, thereby, enabling the plant to bear higher number of pods than the untreated soil condition, and neutralizing soil acidity by lime, which in turn increases availability of P for plant uptake, through reduction in its fixation on acid soils (Kisinyo *et al*., 2016). Lime improve the soil pH, in which enhanced growth and yield of the plant, as a result of increased availability of P that might have increased intensity of photosynthesis, flowering, seed formation and fruiting. Okpara and Muoneke (2007) reported that the application of lime significantly increased number of pods per plant for soybeans. Workneh *et al*. (2013) also reported that application of lime produced the highest seeds per plant than unlimed soil.

The highest hundred seed weight was recorded for JMALM/PR142-15-SC genotype under lime treated soils and the lowest hundred seed weight was recorded for H-7 under lime untreated soil (Table 6). In this study, the variable of tested genotypes has been observed, which indicates the presence of difference among the tested genotypes for hundred seed weight. Application of lime does not affect hundred seed weight of genotypes. In agreement with this result, Habtamu (2017) reported significant difference among soybean genotypes for hundred seed weight, in which the highest hundred seed weight was produced by BFS 39 genotype and the lowest hundred seed weight was recorded from Roba. Bambara and Ndakidemi (2010) reported non-significant effect of liming on hundred seed weight of common bean. In general; lime application to the soil increased yield, number of pod and seed and above ground biomass of soybean genotypes by about 13.67, 16.06, 15.53 and 16.18% respectively, however hundred seed weight were not affected by lime.

Table3: Mean square of growth, root, nodulation, yield and yield components of soybean genotypes grown on limed and unlimed soil on field.

Source of variations	Gen	lime	lime*gen	Error (a)	Total
Parameters					
Yield	582515.8**	341225.8**	98147.32**	4211.35	10148754.36
No of pod per plant	195.62**	239.44**	39.59**	0.41346	3558.5
No of seed per plant	855.29**	801.62**	144.54**	3.444	15005.62
Above ground biomass	5.275**	5.436**	1.3597**	0.1852	109.9
Number of nodule	206.31**	1626.47**	55.06**	1.143	5350.77
Root dry weight	0.093**	0.2722**	0.01715**	0.001716	1.9161
Plant height	810.97**	577.85**	7.4215**	2.143	12190.7
Root volume	1.0074**	4.71512**	0.2408^{ns}	0.227	35.624
Shoot dry weight	7.3028**	40.1735**	2.141**	0.0228	173.756
Hundred seed weight	19.486**	10.64**	0.739^{ns}	3.122	492.733

**= highly significant different at 1 % level of significance, ns= non-significant d/t at 5 % level of significance

Table 4: Interaction effect of genotypes and lime for yield and yield components of soybean genotypes grown under limed and unlimed soil at Mettu during 2017 main cropping season.

Genotypes	Yield (kg/ha)		NPPP		NSPP		AGB(ton/ha)	
	L	UL	L	UL	L	UL	L	UL
PI567046A	1943.93^{a}	1069.87^{d-i}	47.1^{a}	26.6^{cd}	92.33^{a}	55.87^{c}	7.02^{a}	3.23^{e-k}
HAWASSA-04	1576.77^{ab}	1553.11^{bc}	29.36^{bc}	22.93^{gh}	60.73^{b}	41.53^{e-i}	5.06^{b}	4.2^{bc}
JM-PR142/H3-15-SB	1328.29^{b-d}	1027.24^{e-j}	21.53^{h-k}	19.46^{l-n}	43.67^{ef}	37.13^{j-l}	4.24^{bc}	3.58^{c-g}
BRS268	1143.47^{d-g}	1319.83^{b-e}	30.33^{b}	27.6^{cd}	47.73^{d}	47.73^{d}	4.01^{cd}	3.79^{c-f}
JMALM/PR142-15-SC	1214.46^{c-f}	1121.35^{d-g}	20.27^{k-m}	21.33^{i-k}	44.9^{de}	44.67^{de}	4.01^{c-e}	3.96^{c-e}
JM-H3/SCS-15-SG	956.49^{f-k}	1096.45^{d-h}	20.53^{j-l}	20.46^{j-m}	44.36^{de}	43.53^{ef}	3.52^{c-h}	3.57^{c-g}
JM-CLK/G99-15-SB	1076.24^{d-h}	756.98^{j-p}	22.93^{gh}	21.8^{h-j}	38.9^{i-k}	39.13^{h-j}	2.79^{h-m}	2.65^{j-n}
JM-CLK/CRFD-15-SA	935.05^{f-l}	643.49^{m-p}	22.53^{hi}	18.07^{m-o}	40.33^{f-j}	33.47^{l-n}	3.43^{d-j}	2.89^{g-m}
JM-DAV/PR142-15-SA	934.71^{f-l}	915.36^{g-m}	24.33^{fg}	24.8^{ef}	42.76^{e-h}	42.26^{e-i}	2.99^{g-m}	3.07^{f-l}
H-7	772.48^{j-p}	821.79^{h-n}	21.33^{i-k}	18^{nop}	39.67^{g-j}	36.87^{j-l}	2.44^{l-n}	2.27^{m-o}
JM-CLK/G99-15-SC	783.83^{i-o}	818.21^{h-n}	22.53^{hi}	21.93^{h-j}	43.13^{e-g}	43.07^{e-g}	3.51^{c-i}	3.52^{c-h}
SCS-1	618.95^{n-p}	510.49^{p}	17.93^{n-p}	16.53^{p}	32.16^{mn}	29.8^{n}	2.58^{k-n}	2.36^{l-n}
JM-HAR/DAV-15-SA	737.46^{k-p}	690.96^{k-p}	19.27^{l-n}	17.4^{op}	34.33^{lm}	31.87^{mn}	2.35^{l-n}	2.23^{m-o}
JM-ALM/H3-15-SC-1	653.17^{m-p}	637.54^{n-p}	21.27^{i-k}	18.93^{m-o}	39.1^{ijk}	35.27^{k-m}	3.06^{f-l}	2.73^{h-m}
PI423958	682.82^{l-p}	528.21^{o}	13.33^{q}	9.76^{r}	22.13^{o}	14.33^{p}	1.87^{no}	1.43^{o}
Mean	1023.87	900.73	23.63	20.37	44.40	38.43	3.52	3.04
CV	6.74		2.922		4.48		13.12	

Means with the same letters are not significantly different at 5 % level of significance. UL- unlimed; L-Limed; NPPP- Number of pod per plant; NSPP– Number of seed per plant; AGB- above ground biomass; CV- coefficient of variation

Table 5: Average values of Grain Yield (kg/ha), NPPP, NSPP, AGB (ton/ha) and HSW (g) of soybean genotypes grown under limed and unlimed acid soil at Mettu during 2017

Treatments	Yield (kg/ha)	NPPP	NSPP	AGB(ton/ha)	HSW(g)
Limed	1023.87[a]	23.63[a]	44.40[a]	3.525[a]	13.34[a]
Unlimed	900.73[b]	20.36[b]	38.43[b]	3.033[b]	14.34[a]
PR	**13.67**	**16.06**	**15.53**	**16.22**	**-6.97**

Means with the same letters are not significantly different at 5 % level of significance. NPPP-Number of pod per plant; HSW = hundred seed weight, NSPP– Number of seed per plant; AGB-above ground biomass, PR= percent of reduction

Table 6: Main effect of soybean genotypes for hundred seed weight grown under limed and unlimed acid soil at Mettu on field during 2017 main cropping season.

Sub-plot treatments(Genotypes)	HSW (g)
PI423958	17.75[a]
JMALM/PR142-15-SC	16.43[ab]
HAWASSA-04	15.08[bc]
JM-H3/SCS-15-SG	15.078[bc]
JM-PR142/H3-15-SB	14.68[bc]
BRS268	14.46[bc]
JM-CLK/CRFD-15-SA	14.228[c]
JM-HAR/DAV-15-SA	13.83[c]
JM-CLK/G99-15-SB	13.66[c]
JM-CLK/G99-15-SC	13.39[c]
JM-ALM/H3-15-SC-1	13.35[c]
SCS-1	13.31[c]
JM-DAV/PR142-15-SA	13.18[c]
PI567046A	11.08[d]
H-7	10.53[d]
CV	12.61725

Means with the same letters are not significantly different at 5 % level of significance. HSW = hundred seed weight, CV= Coefficient of variation

Effect of Soil Acidity on root, growth and nodulation parameters of soybean genotypes

There were highly significant ($P<0.01$) differences among genotypes for root dry weight, number of nodule, plant height and shoot dry weight in both soils regimes. The interaction of lim ing*genotypes was also highly significant($p\leq0.01$) for root dry weight, number of nodule, plant height and shoot dry weight, except for root volume only the main effect of liming and genotype s were significant. Growth, root and nodulation parameters of soybean genotypes grown under lime treated and untreated soils are indicated in (Table 7&8). On average, the genotypes gave

higher root, growth and nodulation parameters under lime treated acid soil (Table 7 & 8). These results signified that application of lime increasing root, growth and nodulation parameters. JMALM/PR142-15-SC and BRS268 genotype gave higher root volume and root dry weight in both limes treated and untreated soil, and indicating these genotypes might be among acidic soil tolerant genotypes; while genotype PI423958 gave the lowest root dry weigh on lime untreated acid soil (Table 8). Kuswantoro (2015) reported that among the fifteen soybean genotypes tested MLGG 0064 genotype showed the highest root dry weight under the control soil condition (pH 7), while the lowest root length was shown by genotype MLGG 0377 in Mn toxicity condition, which shows varietal difference for acid soil adaptation.

The alteration of root dry weight and root volume includes decreasing and increasing of the root length and root hair. Decrease percentage of root dry weight and root volume in acid soil stress conditions varied among the tested genotypes. Limed soil condition showed the highest root dry weight and root volume than in unlimed soil. This might be due to liming improved the P uptake capacity of plants which facilitate root growth, and then increased root diameter or root thickness of the genotypes, and root dry weight is the result of root growth and development, including root length and number of lateral roots. Alteration in root length and number of roots causes an alteration in root dry weight and root volume. Alteration also occurs on root hair length and root hair density (Haling et al., 2011) that cause an alteration in root dry weight.

There was no negative values existed for root dry weight in unlimed acid soil conditions (Table 6), but negative value existed for number of nodule. Negative value suggests an increasing variable from the optimal or relatively optimal condition to the severer condition. The highest number of nodule was obtained from JM-DAV/PR142-15-SA; while the lowest number of nodule was recorded from SCS1 genotype. This might be due to liming effect on nodule weight and nodule numbers. There was one genotype showing an increase in number of nodule in unlim ed acid soil condition. Abush *et al*. (2017) who reported that, two soybean genotypes i.e., H3 and PR-142 (26) showed the highest number of nodules per plant at 100 kg ha^{-1}P with lime, while, the lowest number of nodules per plant was showed by Essex-1 genotype at 0 kg ha^{-1} p without lime among the other genotypes. The highest plant height was recorded for PI567046A genotype both under lime treated and untreated acid soils. On the other hand, the shortest plant height was recorded for PI423958 genotype under lime untreated soil (Table 7). This indicated that

genotypes responded to liming, which might be due to the effect of liming that neutralized soil acidity, which in turn might have improved the availability of plant nutrients, particularly phosphorus and calcium and lowered the concentration of toxic cations, mainly Al^{3+} ions. The results are similar with the results of Kisinyo *et al*. (2016) who reported that a growth of plant is increased on acid soil in response to the application of lime.

The highest shoot dry weight was obtained from JM-CLK/G99-15-SB genotype, while the lowest shoot dry weight were recorded from SCS-1 genotype (table 7). The reduction of shoot dry weight under the control or unlimed acidic soil condition might be due to Al toxicity, and low Ca, Mg and P concentrations in the shoot, which resulted in decreased photosynthetic capacity that directly affected, shoot growth and developments. This alteration was also due to the low pH inhibits root growth; reduce $Ca^{2+}$and Mg^{2+} in the leaf and reduce rhizobia activity to f orm nodule (Liang *et al*., 2013) as well as the Mn toxicity. Different result was reported; Murata *et al.* (2003) reported that decreasing solution pH and Ca concentration decreased the shoot dry weight. However, plant height, root volume, root dry weight, number of nodule and shoot dry weight is also affected by the genotypes. In general; lime application to the soil increased plant height, root volume, root dry weight, number of nodule and shoot dry weight of soybean ge notypes by about 11.91, 24.47, 17.6, 31.22 and 32.5% respectively.

Table 7: Interaction effect of genotypes and lime for SDW, NN and PHT of soybean genotypes grown under limed and unlimed soil at Mettu during 2017 main cropping season.

Genotypes	SDW (g/plant)		NN/plant		PHT (cm)	
	L	UL	L	UL	L	UL
PI567046A	6.32^{cd}	2.29^{r}	32.56^{f}	20^{m}	83.73^{a}	73.267^{b}
HAWASSA-04	7.045^{ab}	5.83^{d-h}	39.067^{b}	33.6^{e}	55.40^{c}	50.33^{cdef}
JM-PR142/H3-15-SB	5.40^{hij}	3.99^{no}	39.40^{b}	32.27^{fg}	54.72^{cd}	47.00^{efghi}
BRS268	5.93^{defg}	5.95^{def}	31.23^{ghi}	31.26^{ghi}	53.80^{cde}	48.defgh
JMALM/PR142-15-SC	6.16^{cde}	5.45^{ghij}	37.33^{c}	30.87^{hi}	46.27^{fghi}	44.2^{fghijk}
JM-H3/SCS-15-SG	5.58^{fghi}	4.46^{lmn}	35.20^{d}	26.2^{j}	48.93^{cdefg}	44.87^{fghij}
JM-CLK/G99-15-SB	7.56^{a}	4.46^{lmn}	37.33^{c}	23.33^{kl}	43.067^{ghijk}	40.67^{jklm}
JM-CLK/CRFD-15-SA	4.34^{mn}	2.92^{pq}	37.13^{c}	22.67^{l}	48.93^{cdefg}	42.73^{ghijk}
JM-DAV/PR142-15-SA	4.76^{klm}	4.78^{klm}	32.40^{f}	31.67^{fg}	37.33^{klmno}	34.40^{lmno}
H-7	5.18^{ijk}	4.027^{no}	39.00^{b}	34.13^{de}	32.00^{nop}	29.60^{op}
JM-CLK/G99-15-SC	5.73^{efgh}	4.80^{klm}	30.33^{i}	23.2^{kl}	48.8^{cdefg}	43.13^{ghijk}
SCS-1	3.60^{o}	1.97^{r}	24.20^{k}	15.67^{o}	44.83^{fghij}	39.0^{jklmn}
JM-HAR/DAV-15-SA	4.83^{kl}	4.97^{jk}	31.23^{ghi}	30.8^{hi}	38.6^{jklmn}	33.60^{mnop}
JM-ALM/H3-15-SC-1	2.94^{p}	2.38^{qr}	34.36^{de}	17.6^{n}	45.87^{fghi}	41.50^{hijkl}
PI423958	6.48^{bc}	3.69^{o}	55.27^{a}	35.16^{d}	32.39^{nop}	26.20^{p}
Mean	5.45	4.12	35.72	27.22	47.63	42.56
CV	**3.15**		**3.39**		**3.245**	

Means with the same letters are not significantly different at 5 % level of significance. UL- unlimed; L- Limed; SDW- shoot dry weight; NN– number of nodule; PHT- plant height; CV- coefficient of variation

Table 8: Main effect of soybean genotypes for RV and interaction effect of lime and genotypes for RDW grown under limed and unlimed acid soils at Mettu on field during 2017 main cropping season.

Genotypes	RDW (g/plant)		RV /plant
	Limed	Unlimed	
PI567046A	0.75^{cde}	0.433^{j}	2^{bcde}
HAWASSA-04	0.81^{abcd}	0.807^{abcd}	2.33^{bc}
JM-PR142/H3-15-SB	0.79^{bcd}	0.74^{cde}	2.5^{b}
BRS268	0.84^{abcd}	0.83^{abcd}	2.33^{bc}
JMALM/PR142-15-SC	0.937^{a}	0.873^{abc}	3.17^{a}
JM-H3/SCS-15-SG	0.71^{defg}	0.637^{efgh}	2.167^{bc}
JM-CLK/G99-15-SB	0.647^{efgh}	0.44^{j}	1.5^{e}
JM-CLK/CRFD-15-SA	0.893^{ab}	0.75^{cde}	2.43^{b}
JM-DAV/PR142-15-SA	0.73^{def}	0.72^{def}	1.87^{cde}
H-7	0.637^{efgh}	0.58^{ghi}	1.833^{cde}
JM-CLK/G99-15-SC	0.657^{efgh}	0.47^{ij}	1.6^{de}
SCS-1	0.8^{abcd}	0.47^{ij}	2.1^{bcd}
JM-HAR/DAV-15-SA	0.55^{hij}	0.55^{hij}	2^{bcde}
JM-ALM/H3-15-SC-1	0.717^{defg}	0.657^{efgh}	2.033^{bcde}
PI423958	0.59^{fghi}	0.427^{j}	1.833^{cde}
Mean	**0.735**	**0.625**	**CV = 22.55**
CV	**6.093**		

Means with the same letters are not significantly different at 5 % level of significance. RDW- root dry weight; RV– root volume; CV- coefficient of variation

Table 9: Average values of SDW (g/plant), NN/plant, PHT, RDW (g/plant) and RV (g/plant) of soybean genotypes grown under limed and unlimed acid soil at Mettu.

Treatments	SDW (g/plant)	NN/plant	PHT (cm)	RDW (g/plant)	RV /plant
Limed	5.46[a]	35.72[a]	47.64[a]	0.735[a]	2.34[a]
Unlimed	4.12[b]	27.22[b]	42.57[b]	0.625[b]	1.88[b]
PR	**32.52**	**31.23**	**11.91**	**17.6**	**24.46**

Means with the same letters are not significantly different at 5 % level of significance. RDW- root dry weight; RV- root volume, SDW-shoot dry weight, PHT-plant height, NN-number nodule; PR=percent of reduction; CV- coefficient of variation

Table 10: Decrease percentage of yield, above ground biomass, number of pod and seed per plant, number of nodule, root dry weight and plant height of some soybean genotypes under unlimed acid soil conditions.

Genotypes	YLD	AGB	NN	PHT	RDW	NPPP	NSPP
HAWASSA-04	1.50	17.0	13.9	9.13	0.00	21.6	31.6
PI567046A	44.96	54.0	38.4	12.51	42.7	43.4	39.5
PI423958	22.64	23.4	36.5	18.8	27.1	27.4	35.26
JMALM/PR142-15-SC	7.67	1.25	17.3	4.39	7.40	-5.2	0.52
JM-HAR/DAV-15-SA	6.31	5.11	1.39	12.95	0.00	9.69	7.18
JM-PR142/H3-15-SB	22.67	15.5	18.1	14.1	6.30	9.63	14.96
H-7	-6.38	6.84	12.4	7.5	7.90	15.6	7.06
BRS268	-15.4	5.41	-0.1	10.78	1.20	9.03	0.00
JM-H3/SCS-15-SG	-14.6	-1.3	25.5	8.32	11.3	0.36	1.66
JM-CLK/CRFD-15-SA	31.18	15.7	38.9	12.67	15.7	19.8	17.02
JM-ALM/H3-15-SC-1	2.40	10.7	48.6	9.43	8.50	10.9	9.72
JM-CLK/G99-15-SC	-4.39	-0.3	23.5	11.61	27.7	2.66	0.15
SCS-1	17.53	8.41	35.2	13.02	46.3	7.81	7.07
JM-CLK/G99-15-SB	29.66	4.91	37.5	5.52	31.3	4.97	-0.51
JM-DAV/PR142-15-SA	2.06	-2.5	2.26	7.87	2.70	-1.9	1.17

Where, NPPP= number pod per plant, NSPP= number of seeds per plant, PHT= plant height, RDW= root dry weight, YLD= yield, NN= number of nodules per plant, AGB= above ground biomass

Correlation analysis

Grain yield was significantly (P≤ 0.01) and positively correlated with all root parameters viz., root dry weight and root volume and with all growth parameters viz., plant height and shoot dry weight and also with number of nodule at both limed and unlimed soil (Table 7). The significant and positive correlations of grain yield with the rooting parameters viz., root volume and root dry, under acid soil condition or under unlimed acid soil (hydrogen and alumunium toxicity) indicates the importance of the root parameters for acid soil tolerance. This also implies that selection for acid soil tolerance should consider these important root parameters. In line with this result Abush *et al*. (2017) reported that significant and positive associations of soybean grain yield with its root characters viz., root volume, root dry and fresh weight.

Grain yield is the product of its yield components, such as number of pods per plant, number of seeds per plant and above ground dry biomass were highly significant and positively correlated with its grain yield at both lime treated and untreated acid soil (Table 7). However, grain yield was strongly correlated with above ground biomass (r=0.90), followed by number of seeds (r=0.87) and number of pods per plant (r=0.82) at limed soil among yield parameters, respectively. Other authors, such as Ortiz *et al*. (2002) and Abeledo *et al*. (2003) reported that the significant associations of barley grain yield with its yield components. Results obtained in this study on soil treated with lime clearly showed that the remarkable increase in number of pods and seeds per plant, and greatly contributed to increase in grain yield of soybean. The negative correlation of number of nodules with number of seed and pod under unlimed soil (Table 7) indicates the competitiveness of these traits.

Table 11: Pearson correlation analysis for growth, root, nodulation, yield and yield components of soybean genotypes grown under lime treated (1st) and lime untreated (2nd) soil on field at Mettu

	YLD	PHT	NSPP	NPPP	AGB	SDW	RV	RDW	NN
YLD	1								
PHT	0.82**	1							
NSPP	0.87**	0.90**	1						
NPPP	0.82**	0.87**	0.95**	1					
AGB	0.90**	0.91**	0.92**	0.86**	1				
SDW	0.56**	0.21ns	0.31*	0.28^{ns}	0.31*	1			
RV	0.15^{ns}	0.15ns	0.04^{ns}	-0.07^{ns}	0.22^{ns}	-0.08^{ns}	1		
RDW	0.37*	0.38*	0.27^{ns}	0.23^{ns}	0.43**	-0.07^{ns}	0.55**	1	
NN	0.01^{ns}	-0.29^{ns}	-0.25^{ns}	-0.29^{ns}	-0.19^{ns}	0.43**	0.07^{ns}	-0.18^{ns}	1

	YLD	PHT	NSPP	NPPP	AGB	SDW	RV	RDW	NN
YLD	1								
PHT	0.52**	1							
NSPP	0.66**	0.75**	1						
NPPP	0.68**	0.67**	0.92**	1					
AGB	0.76**	0.55**	0.70**	0.67**	1				
SDW	0.63**	-0.13ns	0.24ns	0.35*	0.45**	1			
RV	0.48**	0.19ns	0.21ns	0.23ns	0.51**	0.34*	1		
RDW	0.59**	0.09ns	0.29ns	0.36*	0.61**	0.51**	1.0**	1	
NN	0.39**	-0.36*	-0.15ns	-0.08ns	0.07ns	0.70**	0.27ns	-0.18ns	1

Where, NPPP= number pod per plant, NSPP= number of seeds per plant, PHT= plant, Height, SDW= shoot dry weight, RDW= root dry weight, YLD= yield, NN= number of nodules per plant, AGB= above ground biomass

CONCLUSION

For the conclusion, the observed characters showed a different response in acid soil toxicity. The fifteen genotypes responded differently to acid soil. The observed characters of the sensitive genotypes decreased, while the tolerant genotypes could remain stable or increased. Root dry weight, root volume, number of nodule, plant height, shoot dry weight, grain yield, biomass, number of pod per plant, number of seed per plant, and hundred seed weight under unlimed soil were lower than in limed soil condition. However, not all these characters always decrease in unlimed soil condition. Their increments of grain yield in unlimed soil were found for BRS268 genotype. Genotype of BRS268 was the tolerant genotypes based on the reduction percentage of selected parameters. The increasing root dry weight may due to the result of number of lateral and hair root. Therefore, the use of root dry weight as a criterion in soybean tolerance in low pH should be studied further to ensure the increasing root elongation, number of lateral roots and nutrients uptake to support its tolerance, which contributes for yield increments. These results also give a clear indication that the grain yield was very closely associated with number of pods

per plant, seed per plant, root dry weight, root volume, and shoot dry weight and number of nodule in both unlimed and limed soil. It seems that these parameters are useful characters to select for high yield in soybean breeding programs for soil acidity tolerance.

Conflict of Interests

Authors have declared that no competing interests exist.

Acknowledgement

The authors are grateful to the financial grant of the Ethiopian institute of agricultural research through the Jimma agricultural research center. We also acknowledges Jimma agricultural research center for facilitating the research work and also acknowledge the staff members of Mettu research sub center for their technical assistance during the time of conducting the field experiment and data collection.

Credit authorship contribution statement

Tolessa Ameyu: Conceptualization, Methodology, Software, Formal analysis, Investigation, Data curation, Writing - original draft, Writing - review & editing, Visualization. **Abush Tesfaye**: Conceptualization, Methodology, Software, Formal analysis, Investigation, Writing - review & editing, Supervision, Project administration, funding acquisition. **Alemayehu Regassa**: Conceptualization, Methodology, Writing - review & editing, Visualization, Supervision, Project administration, funding acquisition.

REFERENCES

Abush T., M. Githiri, J. Derera and D. Tolessa, 2017. Genetic variability in soybean (*Glycine max L.*) for low soil phosphorus tolerance. *Ethiopian Journal of Agricultural Sciences,* **27:**1-15.

Abeledo G., F. Calderini and A.Slafer, 2003. Genetic improvement of barley yield potential and its physiological determinants in Argentina (1944–1998). *Euphytica,* **130:**.325-334.

Anetor O. and E. Akinrinde, 2006. Response of soybean [*Glycine max (L.) Merrill*] to lime and phosphorus fertilizer treatments on an acidic alfisol of Nigeria. *Pakistan Journal of Nutrition,* **5:** 286-293.

Bambara S. and A. Ndakidemi, 2010. Phaseolus vulgaris response to Rhizobium inoculation, lime and molybdenum in selected low pH soil in Western Cape, South Africa. *African Journal of Agricultural Research*, **5:** 1804-1811.

Baruah C. and P. Barthakur, 1997. *A Textbook of Soil analysis*, Vikas Publishing House, New Delhi, India.

Black A., 1965. *Methods of soil analysis*. Part I, American Society of Agronomy. Madison, Wisconsin, USA. 1572p.

Bouyoucos J., 1962. Hydrometer Method Improved for Making Particle Size Analyses of Soils 1. *Agronomy journal*, **54:** 464-465.

Brady C. and R. Weil, 2002. The nature and properties of soils, 13th. *Pearson education (Singapore) Pte. Ltd. Indian Branch*, **482**: 621-624.

Bray H. and T. Kurtz, 1945. Determination of total, organic, and available forms of phosphorus in soils. *Soil science*, **59:**39-46.

Champman D., 1965. Cation exchange capacity by ammonium saturation. In: black, C.A., L.E., Ensminger and F.E., Clark (Eds.). Method of soil analysis. *American Society of Agronomy*. Madison Wisconsin, USA. PP. 891- 901.

Delhaize E., D. Gruber and R. Ryan, 2007. The roles of organic anion permeases in aluminium resistance and mineral nutrition. *Febs Letters*, **581:** 2255-2262.

Fageria K. and C. Baligar, 2008. Ameliorating soil acidity of tropical Oxisols by liming for sustainable crop production. *Advances in agronomy*, **99:** 345-399.

Haling E., J. Simpson, A. Culvenor, H. Lambers and E. Richardson, 2011. Effect of soil acidity, soil strength and macropores on root growth and morphology of perennial grass species differing in acid-soil resistance. *Plant, Cell & Environment*, **34:** 444-456.

Kisinyo O., 2016. Long term effects of lime and phosphorus application on maize productivity in an acid soil of Uasin Gishu County, Kenya. *Sky Journal of Agricultural Research*, **5:** 48 - 55.

Kuswantoro H, 2015. Tolerance of fifteen soybean germplasm to low pH condition. *Intl J Plant Breed Genetics* **9:** 189-197.

Liang C., A. Pinero's, J. Tian, Z. Yao, L. Sun, J. Liu, J. Shaff, A. Coluccio, V. Kochian and H. Liao, 2013. Low pH, aluminum, and phosphorus coordinately regulate malate exudation through GmALMT1 to improve soybean adaptation to acid soils. *Plant Physiology*, **161:** 1347-1361.

McLean O., 1965. Aluminum. In: C.A. Black (ed.), Methods of Soil Analysis. Agronomy, No.9. Part II. American Society Agronomy, Madison, Wisconsin. USA, pp 978 - 998.

Mesfin A., 2007. Nature and management of acid soils in Ethiopia. *Addis Ababa, Ethiopia.* 99p.

Murata R., S. Hammes and E. Zharare, 2003. Effect of solution pH and calcium concentration on germination and early growth of groundnut. *Journal of plant nutrition*, **26:** 1247-1262.

Okpara D and C. Muoneke, 2007. Influence of lime on the performance of high-yielding soybean varieties in southern Nigeria. *Agro-Science journal*. ISSN: pp. 1119-7455

Ortiz-Monasterio P., H. Pfeiffer and H. Hede, 2002. Phosphorus use efficiency, grain yield and quality of triticale and durum wheat under irrigated conditions In: Proceedings of the 5th International Symposium, Annex, Radizkow, Poland.

SAS (Statistical Analysis System) soft ware, 2012. Version9.3, SAS institute, Cary, NC, USA

Shahid Q., F. Saleem, Z. Khan and A. Anjum, 2009. Performance of soybean (*Glycine max* L.) under different phosphorus levels and inoculation. *Pakistan Journal of Agricultural Sciences*, **46:** 237 - 241.

Shoemaker E., O. Mclean and F. Pratt, 1961. Buffer methods for determing lime requirement of soils with appreciable amounts of extractable aluminum 1. *Soil Science Society American*. Proceeding, **25:** 274 - 277.

Shoemaker E., O. Mclean and F. Pratt, 1961. Buffer methods for determing lime requirement of soils with appreciable amounts of extractable aluminum 1. *Soil Science Society American*. Proceeding, **25:** 274 - 277.

Temasgen D., G. Alemu, A. Adella and D. Tolessa, 2017. Effect of lime and phosphorus fertilizer on Acid soils and barley (*Hordeum vulgare l.*) performance in the central highlands of Ethiopia. *Experimental Agriculture*, **53:** 432-444.

Von Uexkull R and E. Mutert, 1995. Global extent, development and economic impact of acid soils. *Plant and soil*, **171:**1-15.

Wang P., H. Raman, P. Zhang, N. Mendham and X. Zhou, 2006. Aluminium tolerance in barley (*Hordeum vulgare L.*): physiological mechanisms, genetics and screening methods. *Journal of Zhejiang University SCIENCE B*, **7:**769-787.

Click here to access/download
Supporting Information
List of table PLOS ONE.doc
