# Supplementary material for: Tolerance to soil acidity of soybean (Glycine max L.) genotypes under field conditions Southwestern Ethiopia
Source: PLoS One. 2022 Sep 15;17(9):e0272924. doi: 10.1371/journal.pone.0272924 (PMC9477287; doi:10.1371/journal.pone.0272924)
Supplement: Attachment — Submitted filename: Response to reviewer 2.docx [file pone.0272924.s004.docx]

Date: August, 2021

To:”PLOS ONE”plosone@plos.org

From:”Tolessa Ameyu Bedessa” [tolamboo@gmail.com](mailto:tolamboo@gmail.com)

Subject: Authors; Response to reviewer [PONE-D-20-38977]

Dear Editor-in Chief

We are very grateful to the reviewers for these valuable and constructive comments to our manuscript entitled” Tolerance to soil acidity of soybean (Glycine max L.) genotypes under field conditions at Southwestern Ethiopia “PONE-D-20-38977”. We have attempted to address all comments in this revised manuscript and now the manuscript is greatly improved. We highlighted yellow color on the “revised manuscript with track change” for highlights additional inputs and changes made to the original version. The responses given for comments are highlighted red color in author’s response page. Below are our responses to the specific comments raised by the reviewer #3 and #5 and also the response given to reviewer #1 and #2 is kept as it is.

Response to question raised

1. Please review your reference list to ensure that it is complete and correct. If you have cited papers that have been retracted, please include the rationale for doing so in the manuscript text, or remove these references and replace them with relevant current references. Any changes to the reference list should be mentioned in the rebuttal letter that accompanies your revised manuscript. If you need to cite a retracted article, indicate the article’s retracted status in the References list and also include a citation and full reference for the retraction notice.

I have checked all listed references and all references are complete and cited appropriately

2. Please amend your current ethics statement to confirm that your named institutional review board or ethics committee specifically approved this study. Once the title of the manuscript is approved at the initial/starting of the experiment no need of commite to approve for publication because of the authors have full right to publish else were without any permission of the others/ any approval committee. In all no committee to approve this paper

**Reviewer #3**

Manuscript has been poorly written and lacks coherence. Line N0.42 rewrite the sentence, sentence is long: too long sentences raised on line No 42 is corrected with short and precise words. And also the issue raised on coherence is touched.

Line No 60- 63 rewrite the para.

The paragraph is corrected as reviewer comments
Line No. 65 (Abush T) delete T.

The word T were deleted
Line No. 70 replace the word development by identification of .

The word development were replaced by identification of
Line No.71- 73 Sentence is too long and should be modified

The long sentences in modified to clear and short

Line No. 84 Delete the sentence “According to the weather data obtained from the meteorological station of Mettu Agricultural Research Sub Center”. Sentences were deleted

Line No. 125 replace amounts with amount.

The word amounts in replaced by amount
Line No. 134 delete was.

The word **was** were deleted
Line No. 148 root volume.

The word Root is corrected as Root Volume

Line No. 151, Not clear, rewrite the sentence.

The sentences were rewritten clearly as suggested by reviewer

Line No. 184Replace the sentence as, Genotypes reflected significant differences for number of pods----------and above ground biomass in both limed and un limed soils.

The sentences were replaced as the suggestion of the reviewer

Line No.190-191. The sentence doesn’t give any sense and should be deleted.

The senseless sentences were deleted

**Reviewer #5**

The abstract is well written and organized. line 17, “necessary” stage is not correct wording. I suggest to use more suitable word. Separate “BRS268had” into BRS268 had. The word were separated as suggested by reviewer

Please state clearly the novelty of the study and tested hypothesis at the end of the introduction. At the end of introduction tested hypothesis is clearly stated based on reviewer comments

Line 222: The highest HSW recorded was for PI423958 genotype not JMALM/PR142-15-SC genotype. YES thank you …… I correct it so
5. Table 6 is incomplete. Show HSW for lime and un limed or remove limed from the title if the limed treatments did not show any significant effect on HSW. The word limed is removed from the title because of no significant effect for HSW

The paper fails to highlight the significant of the study and the conclusion needs to be expanded significantly to explain clearly about the novelty of the experimental setup. Also, policy recommendations can be suggested. Coclussion were modified and also the best recommendation and future direction is clearly mentioned after the suggestion of the reviewer

**Reviewer #1**

It would be very helpful if the authors would include continuous line numbers to facilitate the review process. Continuous line number is included throughout the document based on the comment of reviewer #1

The authors also need to provide the raw data used in the analyses in supplementary files. The following observations may also addressed adequately while revising the manuscript. Under supplementary files the raw data of all included parameter is provided based on the comment of reviewer#1

For some of the experiments, the authors use 5 random plants of each genotype. This is an extremely limited amount of sampling given that they are drawing conclusions that apply to the genotype. Because of the experimental plot size were low (4m by 2.4 m (9.6 m^2^), which is two harvestable rows the sample taken is enough to represent this plot treatment.

There are also areas where the authors need to provide citations for the statements they make. The citation missed at necessary place is included and highlighted with yellow color, both in introduction, methodology and discussion part of the manuscript.

In several places, the work done has been unfortunately obscured by very poor English language communication. It is highly recommended that the authors seek the help of English language editors in order to improve the quality of the manuscript. In some part of this manuscript we was try to improve or replaced unclear English words with the correct or appropriate words.

**Reviewer #2**

Comment: the abstract of manuscript entitled” Tolerance to soil acidity of soybean (Glycine max L.) genotypes under field conditions at Southwestern Ethiopia, can serve as a stand-alone document, which succinctly described. So, the abstract should cover the salient findings more critically. Some sentences are too long in abstract, methodology and results and discussion. Describe it briefly or split it another sentence if possible.

The sentences those too long in abstract, methodology and result and discussion are described separately with short and brief sentences as reviewer comments (highlighted in manuscript in yellow color)

Comment: The introduction is informative, precise, and comprised of relevant content. The literary structure of the introduction is also good. Citation is missing at the end of paragraph 1, 3, 4, etc. The citation missed at the end of listed paragraph is included accordingly. (Takala, B., 2019). (Tesfaye, A., 2017). (Abush T *et al*.,2017 and *Adisu et al., 2019*).

Comment: Material and methods section looks fine, the used methods are well presented. The experimental design was appropriate and the applied protocols were used correctly. The authors organized very nice research. The methods are simple and sufficiently described. However, gave proper reference that from the soybean genotypes identified previously. The proper source from which each genotypes is identified described in table 2 of the manuscript.

Comment: In Table 3. What does mean Gen? Specify it/mention if used it as abbreviated. Gen represents the word genotypes in this manuscript and this comment is mentioned in the body of manuscript.

Comment: Why Average values of hundred seed weight is higher in un-limed soil. Justify it. Gave it reason in discussion section.
Hundred seed weight is higher in un-limed soil, this might be under limed acid soil there is the improvement of soil pH in response to lime amendment, which enhanced growth and yield of the plant, as a result of increased availability of P that might have increased intensity of photosynthesis, flowering, seed formation and fruiting(forming high number of pod and seed), as a result these formed fruit/seed is competed for nutrient to fulfill the seed and the seed size become decreased which have the direct effect on seed weight. (in other way under unlimed acid soil the number of pod and fruit formed is low and less competition for nutrition)

Comment: Either it will be better to analyze the data statistically separately lime treated and un-limed soil, instead of combine? To see the interaction effect of genotypes and soil amendments it is good if we analyses in combine than separate.

Comment: In this paper you have just mentioned and analyzed toxicity of H+ and Al3+, Is there no any else toxicity i.e., Fe, Cu, Zn, Mn, etc., as common problem found in acidic soils. Answer: there is a problem of Fe, Cu, Mn in acid soil, however the status of Ethiopian soil were accessed by Ethiopian agricultural transformation agency (ATA) and identified H+ and Al3+ cations as a major problem than the rest of acid cation and also as we observed the soil of the study area from previous experiment no other acid cation problem at specific location than H+ and Al3+ cations

Comment: The discussion section is also well described. Discussion should be appropriate in which results are discussed critically in response to support as well contradiction, instead of describing other cited results. Justify your results with some latest citations.

The result is justified with latest citation and highlighted in yellow color in the body of this manuscript.
Comment: Conclusions is presented in an appropriate fashion however, looking too long. To long conclusion is presented with short and precise form.

Comment: On what basis, Genotype BRS268 showed higher yield (1319.83 kg ha-1 ) under lime untreated acid soil than lime treated acid soil(1143.47 kg ha -1 ) and showed less reduction percentage for number of nodule, root weight and number of seeds per plant? Either it resistant? Is it previously identified/claimed? Yes it is previously identified as resistant to acid soil by Brazilian breeder and soil scientist.
