# Supplementary material for: Tolerance to soil acidity of soybean (Glycine max L.) genotypes under field conditions Southwestern Ethiopia
Source: PLoS One. 2022 Sep 15;17(9):e0272924. doi: 10.1371/journal.pone.0272924 (PMC9477287; doi:10.1371/journal.pone.0272924)
Supplement: Attachment — Submitted filename: Response to reviewer.doc [file pone.0272924.s006.doc]

Date: April 2022

To:” PLOS ONE” plosone@plos.org

From:”Tolessa Ameyu Bedessa” [tolamboo@gmail.com](mailto:tolamboo@gmail.com)

Subject: Authors; Response to reviewer [PONE-D-20-38977]

Dear Editor-in-Chief

We are very grateful to the reviewers for these valuable and constructive comments to our manuscript entitled” Tolerance to soil acidity of soybean (Glycine max L.) genotypes under field conditions at Southwestern Ethiopia “PONE-D-20-38977”. We have attempted to address all comments in this revised manuscript and now the manuscript is greatly improved. We highlighted yellow color on the “revised manuscript with track change” for highlights additional inputs and changes made to the original version. The responses given for comments are highlighted in red color in the author’s response page. Below are our responses to the specific comments raised by reviewers.

Response to question raised by the reviewer highlighted by red color

**The latest response to the reviewer
Reviewer#7** suggested adding articles entitled “Abderrahmane et al. Influence of Highway Traffic on Contamination of Roadside Soil with Heavy Metals”, “Nkansah et al. Preliminary Studies on the Use of Sawdust and Peanut Shell Powder as Adsorbents for Phosphorus Removal from Water” and “J. Sam. Compressive Strength of Concrete using Fly Ash and Rice Husk Ash: A Review” to the literature review: Answer: it is not mandatory to add these authors finding, because of there are too many recent relevant finding which was already cited in the paper.

For readers to quickly catch your contribution, it would be better to highlight major difficulties and challenges, and your original achievements to overcome them, in a clearer way in abstract and introduction. I did it and already highlighted

Some key parameters are not mentioned. The rationale on the choice of the particular set of parameters should be explained with more details. Have the authors experimented with other sets of values? What are the sensitivities of these parameters on the results? All important parameters which are important for acid soil toxicity tolerant selections are mentioned in the paper.i.e., root volume, root fresh and dry weight. For soil acidity tolerant screening genotypes, the well-known parameters are Root data (international experience and scientifically justified). The sensitivity of root data is mentioned in the discussion session.

Please avoid reference overkill/run-on, i.e., do not use more than 3 references per sentence. There are no more than three references per on sentences.

The research contributions of the paper should be articulated more clearly. The abstract is not representative of the content and contributions of the paper. The abstract does not seem to properly convey the rigor of research. Research contribution is sounded clearly, you can see in the introduction part. (It contributes for low income/resource poor farmers to produce soybean without use of liming under acid soil by using acid tolerant varieties). The abstract of the paper is amended according to the comment of reviewer, it explains the content, result and finding of the papers and PLOS ONE regulations.

- Aside from the aim stated in the title, the research gap and the goals of the research are not specified which leads to the reader missing the significance of the research. The research gap is already stated in the background of the paper (see starting from line 69)

This raises some concerns regarding the potential overlap with authors previous works.

My previous work was dealing with testing genotypes under application of phosphorus nutrition treated and untreated

In introduction, add a statement to highlight the research gap/novelty of the study prior to objectives. This is included in introduction prior to objectives based on your comments.
